# Health system-scale language models are all-purpose prediction engines

**DOI:** 10.1038/s41586-023-06160-y

**Published:** 2023-06-07

**Authors:** Lavender Yao Jiang, Xujin Chris Liu, Nima Pour Nejatian, Mustafa Nasir-Moin, Duo Wang, Anas Abidin, Kevin Eaton, Howard Antony Riina, Ilya Laufer, Paawan Punjabi, Madeline Miceli, Nora C. Kim, Cordelia Orillac, Zane Schnurman, Christopher Livia, Hannah Weiss, David Kurland, Sean Neifert, Yosef Dastagirzada, Douglas Kondziolka, Alexander T. M. Cheung, Grace Yang, Ming Cao, Mona Flores, Anthony B. Costa, Yindalon Aphinyanaphongs, Kyunghyun Cho, Eric Karl Oermann

**Affiliations:** 1 Department of Neurosurgery, NYU Langone Health, New York, NY USA; 2 Center for Data Science, New York University, New York, NY USA; 3 Electrical and Computer Engineering, Tandon School of Engineering, New York, NY USA; 4 NVIDIA, Santa Clara, CA USA; 5 Predictive Analytics Unit, NYU Langone Health, New York, NY USA; 6 Department of Internal Medicine, NYU Langone Health, New York, NY USA; 7 Department of Population Health, NYU Langone Health, New York, NY USA; 8 Prescient Design, Genentech, New York, NY USA; 9 Courant Institute of Mathematical Sciences, New York University, New York, NY USA; 10 Canadian Institute for Advanced Research, Toronto, Ontario Canada; 11 Department of Radiology, NYU Langone Health, New York, NY USA

**Keywords:** Translational research, Computational science

## Abstract

Physicians make critical time-constrained decisions every day. Clinical predictive models can help physicians and administrators make decisions by forecasting clinical and operational events. Existing structured data-based clinical predictive models have limited use in everyday practice owing to complexity in data processing, as well as model development and deployment^1–3^. Here we show that unstructured clinical notes from the electronic health record can enable the training of clinical language models, which can be used as all-purpose clinical predictive engines with low-resistance development and deployment. Our approach leverages recent advances in natural language processing^4,5^ to train a large language model for medical language (NYUTron) and subsequently fine-tune it across a wide range of clinical and operational predictive tasks. We evaluated our approach within our health system for five such tasks: 30-day all-cause readmission prediction, in-hospital mortality prediction, comorbidity index prediction, length of stay prediction, and insurance denial prediction. We show that NYUTron has an area under the curve (AUC) of 78.7–94.9%, with an improvement of 5.36–14.7% in the AUC compared with traditional models. We additionally demonstrate the benefits of pretraining with clinical text, the potential for increasing generalizability to different sites through fine-tuning and the full deployment of our system in a prospective, single-arm trial. These results show the potential for using clinical language models in medicine to read alongside physicians and provide guidance at the point of care.

## Abstract (web summary)

A clinical language model trained on unstructured clinical notes from the electronic health record enhances prediction of clinical and operational events.

## Main

Physicians make difficult decisions every day requiring the integration of a tremendous amount of information. The information needed to make these medical decisions is scattered across various records, for example, a patient’s medical history and laboratory and imaging reports. When physicians perform their work, however, all of this information is ultimately integrated into the notes written by physicians to document and summarize patient care.

Clinical predictive models are frequently derived from rules that have existed for decades^6–9^, as well as from machine learning methods^10–12^, with most relying on structured inputs pulled from the electronic health record (EHR) or direct clinician inputs. This reliance on structured inputs introduces complexity in data processing, as well as in model development and deployment, which in part is responsible for the overwhelming majority of medical predictive algorithms being trained, tested and published, yet never deployed to assess their impact on real-world clinical care. This is frequently referred to as the ‘last-mile problem’ (refs. ^1–3^).

One of the most exciting recent developments in modern artificial intelligence (AI) research is large language models (LLMs). These massive neural networks (with millions or even billions of parameters) have been shown to obtain impactful results on a wide range of problems that rely on the reading and interpretation of human language. Several styles of LLMs have been developed over the past few years, broadly ranging from encoder models (such as BERT^4^) to decoder models (such as GPT3; ref. ^5^). We theorized that LLMs could potentially solve the last-mile problem in medical predictive analytics by simply reading the notes written by physicians, thereby immediately accessing a comprehensive description of a patient’s medical state to provide decision support at the point of care across a wide range of clinical and operational tasks.

Here we present our results from developing, evaluating, deploying and prospectively assessing NYUTron, an LLM-based system that can integrate in real time with clinical workflows centred around writing notes and placing electronic orders. Our approach relies on the fact that all clinically useful data and medical professionals’ decision-making processes can be found as structured or unstructured text in the EHR (for example, as notes, laboratory results and reports on studies). Our approach leverages recent advances in natural language processing that suggest that sufficiently scaled, self-supervised LLMs can outperform strongly supervised approaches on non-medical predictive tasks^4,5,13^. We investigate our hypothesis in the NYU Langone Health System (‘NYU Langone’), a large multi-borough hospital system with a diverse patient population in New York, with 4 urban hospitals and 350 outpatient sites. We assess NYUTron on a battery of five tasks, including three clinical and two operational tasks (30-day all-cause readmission prediction, in-hospital mortality prediction, comorbidity index prediction, length of stay (LOS) prediction and insurance denial prediction) and provide a detailed analysis of our 30-day readmission task to look at questions of data efficiency, generalizability, deployability and potential clinical impact. By rethinking all of medical predictive analytics (see Supplementary Information section 1.1 for previous works) as a natural language processing problem, we show that it is possible to use LLMs as universal prediction engines for a wide range of medical predictive tasks.

## Language model-based clinical prediction

**Fig. 1:**
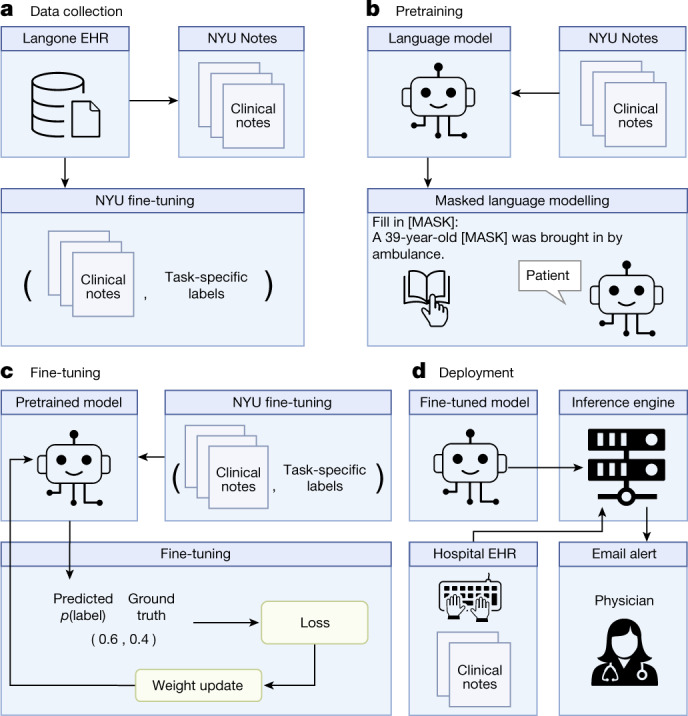
Overview of the language model-based approach for clinical prediction. **a**, We queried the NYU Langone EHR for two types of datasets. The pretraining dataset, NYU Notes, contains 10 years of inpatient clinical notes (387,144 patients, 4.1 billion words). There are five fine-tuning datasets. Each contains 1–10 years of inpatient clinical notes (55,791–413,845 patients, 51–87 million words) with task-specific labels (2–4 classes). **b**, We pretrained a 109 million-parameter BERT-like LLM, termed NYUTron, on the entire EHR using an MLM task to create a pretrained model for medical language contained within the EHR. **c**, We subsequently fine-tuned the pretrained model on specific tasks (for example, 30-day all-cause readmission prediction) and validated it on held-out retrospective data. **d**, Lastly, the fine-tuned model was compressed into an accelerated format and loaded into an inference engine, which interfaces with the NYU Langone EHR to read discharge notes when they are signed by treating physicians.

Our language model-based approach has four steps: data collection, pretraining, fine-tuning and deployment. In the first step (Fig. 1a), we collected a vast set of unlabelled clinical notes and five task-specific labelled clinical notes from the NYU Langone EHR. Unlike other studies, our datasets come from the entire hospital system with a diverse patient population from different clinical departments. Our large unlabelled dataset, ‘NYU Notes’, comprises 7.25 million clinical notes (for example, radiographic reads, history and physicals) from 387,144 patients across four hospitals, resulting in a 4.1 billion-word corpus curated from January 2011 to May 2020. Each one of our labelled fine-tuning sets contains 1–10 years of inpatient clinical notes (55,791–413,845 patients, 51–87 million words) with task-specific labels (2–4 classes). See Extended Data Table 1 for dataset statistics.

In the second and third steps (Fig. 1b,c), we pretrained and fine-tuned an LLM for each downstream task using a bidirectional encoder model known as BERT (Bidirectional Encoder Representation with Transformer) and a masked language modelling (MLM) objective on the NYU Notes dataset^11^ until the validation loss plateaued. The MLM objective randomly masks words or subwords in clinical notes and trains the language model to fill in the masked word correctly. Next, using the fine-tuning dataset, we fine-tuned the pretrained model (termed ‘NYUTron’) to predict the task label using the relationships learned in pretraining with clinical notes.

In the fourth step (Fig. 1d), we deployed our best model to a high-performance inference engine, NYUTriton, that interfaces with the NYU Langone EHR. Deployment enabled real-time LLM-guided inference at the point of care. In a single-arm, non-interventional, prospective trial, we validated NYUTron’s performance on 30-day readmission prediction in a real-world environment and assessed its potential clinical impacts.

## Overall performance on five tasks

**Fig. 2:**
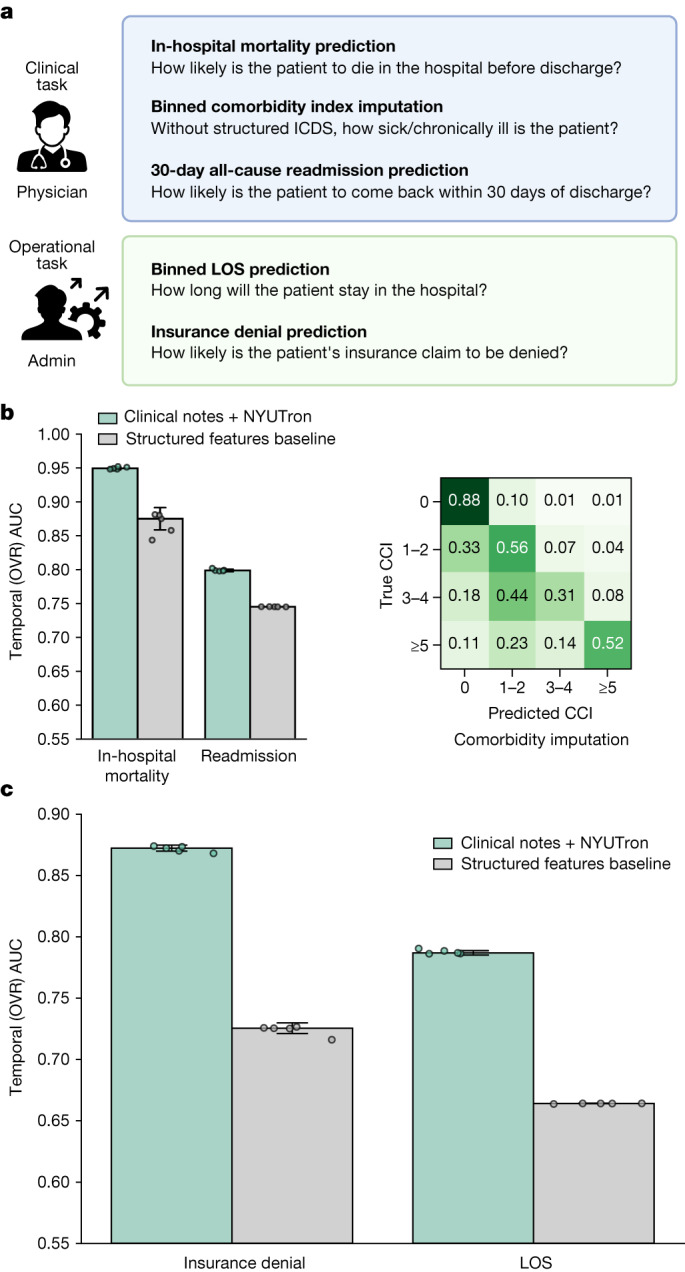
Overall temporal test performance across five tasks. **a**, The five tasks include three clinical tasks and two operational tasks. **b**, On readmission prediction, NYUTron had a median AUC of 79.9% ± 0.168% with a 5.36% improvement. On in-hospital mortality prediction, NYUTron had a median AUC of 94.9% ± 0.168% with a 7.43% improvement. On comorbidity index imputation, NYUTron had an OVR median AUC of 89.4% ± 0.275%. A confusion matrix is shown on the right. **c**, On binned LOS prediction, NYUTron had a median AUC of 78.7% ± 0.179% with a 12.3% improvement from the structured baseline. On insurance denial prediction, NYUTron had a median AUC of 87.2% ± 0.246% with a 14.7% improvement. For **b**,**c**, the height of the error bar is the median AUC and the half-width of the error bar is 1 s.d. The grey points are individual data points from *n* = 5 experiments using distinct random seeds.

To assess the breadth of NYUTron’s applicability, we evaluated NYUTron’s performance on five tasks retrospectively. We trained with the full dataset and evaluated performance with two test sets: (1) a random test set (clinical notes sampled from the same time as the training data) and (2) a temporal test set (clinical notes sampled from the future of the training data). The temporal test set more closely resembles the deployment scenario, in which inference data come from the future of the training data. Our battery of tasks consisted of three clinical tasks and two operational tasks, as shown in Fig. 2a. We compared NYUTron against structured baselines, which forward structured features used by traditional clinical predictive models into an extreme gradient-boosted tree^14^ model.

NYUTron is capable of being extended to multiple clinical and operational tasks. Figure 2b and Fig. 2c show that, on prediction tasks (in-hospital mortality, readmission, LOS and insurance denial), NYUTron had an area under the curve (AUC) of 78.7–94.9%, with an improvement of 5.36–14.7% in AUC from traditional clinical predictive models. On the comorbidity index imputation task, NYUTron had a median AUC of 89.4% ± 0.275%. We first present our results across four of the tasks and conclude with a focused look at readmission prediction that addresses questions of data efficiency, model generalizability and deployment in a real-world environment.

NYUTron is capable of predicting risk of in-hospital mortality on admission and imputing a comorbidity index. The task of in-hospital mortality prediction was to estimate (at admission) the likelihood of a patient’s death during the present inpatient encounter. Figure 2b shows that, for in-hospital mortality prediction, NYUTron had a median AUC of 94.9% ± 0.168%, with a 7.43% improvement from its structured baseline based on Simplified Acute Physiology Score (SAPS2)^15^ and Acute Physiology and Chronic Health Evaluation (APACHE2)^16^ features such as age and mean heart rate. The task of comorbidity index imputation was to predict (at admission) the Charlson comorbidity index (CCI)^17^ with no available structured features for chronic diseases. We framed this as a data imputation problem, as 22% of our dataset lacked CCI scores and this was a known area for documentation improvement (see Supplementary Information section 2.3 for more context). We discretized the index into four bins according to the original paper’s grades of severity (0, none; 1–2, mild; 3–4, moderate; ≥5, severe). Figure 2b shows that, on comorbidity imputation, NYUTron had a median AUC of 89.4% ± 0.275% and 88% precision when identifying patients whose CCI score was 0.

NYUTron can also be used for operational endpoints and to predict in-patient LOS and insurance claim denial on admission. The task of LOS prediction was to predict (at admission) the likely range of days a patient would stay in the hospital. We discretized LOS into four bins (0–25% quantile, 25–50% quantile, 50–75% quantile, >75% quantile). Figure 2c shows that, for LOS prediction, NYUTron had a median one-versus-rest (OVR) AUC of 78.7% ± 0.179%, with a 12.3% improvement from the structured baseline, which used an available subset of ‘Lisbon Portugal’ features^18^. The task of insurance claim denial prediction was to predict (at admission) whether the insurance claims submitted for an encounter would be accepted or initially denied. Figure 2c shows that, for insurance denial prediction, NYUTron had a median AUC of 87.2% ± 0.246%, with a 14.7% improvement from the structured baseline, which used an available subset of ‘claim form’ features^19^ such as age and insurance provider. NYUTron is also capable of predicting different types of denials from both admission notes and discharge notes with similar performance (Supplementary Information section 2.2).

## Detailed analysis on readmission

To better understand NYUTron’s performance, we carried out a detailed analysis of 30-day all-cause readmission prediction. The task of readmission prediction is to predict (at discharge) the likelihood of a patient coming back to the hospital within 30 days and is a well-studied problem in the medical informatics literature (see Supplementary Information section 2.1 for more details on the readmission prediction task). Figure 2b shows that, for 30-day all-cause readmission prediction, NYUTron had a median AUC of 79.87% ± 0.168%, with a 5.36% improvement from its structured baseline, which used LACE^20^ features (a mnemonic for LOS, acuity of admission, Charlson comorbidity index and number of emergency department visits in the past 6 months). We performed five additional evaluations in both retrospective and prospective settings: (1) a human comparison with six attending physicians for prediction of readmission for 20 patient cases sampled from a random split, (2) a study of NYUTron’s scaling properties with respect to data in which NYUTron and other models were compared using a different number of fine-tuned data points, (3) an assessment of NYUTron’s cross-site generalizability using pretraining, fine-tuning and test data from different locations, (4) a prospective, single-arm, non-interventional study to evaluate NYUTron’s deployability and (5) a qualitative evaluation by a physician panel of NYUTron’s prospective performance to assess clinical impacts.

## Retrospective study of readmission

**Fig. 3:**
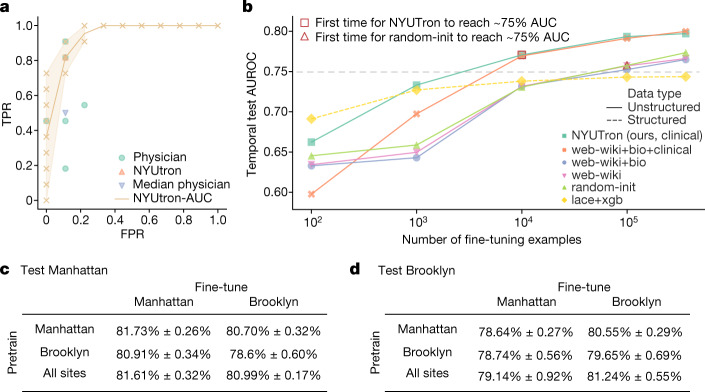
Retrospective study of NYUTron’s readmission prediction. **a**, On 20 cases sampled from a random split, we compared NYUTron’s TPR and FPR with those for six physicians. NYUTron (orange triangles) had a higher TPR and the same FPR when compared with the median physician performance (green circles). The error band for AUC ranges from the minimum to maximum, and the orange crosses indicate TPR and FPR using all possible thresholds. We chose NYUTron’s threshold on the basis of validation data. **b**, Comparison of the temporal test AUCs of different pretrained LLMs with an increasing number of fine-tuning examples. For simplicity, we omit the variance and only plot the median performance of five trials. Differences in median performance with 100 and 1,000 examples are less notable because AUCs with sparse fine-tuning examples have high variance (at 100 examples, we had 4.26% to 9.56% variance; at 1,000 examples, we had 0.44% to 9.46% variance). AUC variance decreases with more fine-tuning examples. The horizontal dashed line at 0.75 corresponds to the threshold for performance. See alternative presentations in Extended Data Fig. 7. **c**,**d**, Temporal test performance of NYUTron using pretraining, fine-tuning and test data from different sites. For both the Manhattan and Brooklyn tests, the column corresponding to local fine-tuning shows better performance than that with external fine-tuning. Each entry in **c**,**d** is presented as the mean ± 1 s.d. for *n* = 5 experiments using distinct random seeds.

On small samples, NYUTron was competitive with a small group of physicians at predicting 30-day readmission. We tested a group of six physicians at different levels of seniority against NYUTron in a head-to-head comparison to establish a baseline difficulty for predicting 30-day all-cause readmission at the time of discharge. Discharge summaries (*n* = 20, including 11 positive cases and 9 negative cases) were sampled from a random split and uploaded to an online evaluation platform. Median physician performance was worse than that of NYUTron (Fig. 3a). For physicians and NYUTron, the median false positive rate (FPR) was 11.11%, whereas the median true positive rate (TPR) was 50% for physicians compared with 81.82% for NYUTron. Physicians had a median F1 score of 62.8% and substantial variance of 22.2% compared with NYUTron, which had a median F1 score of 77.8%.

**Extended Data Fig. 1:**
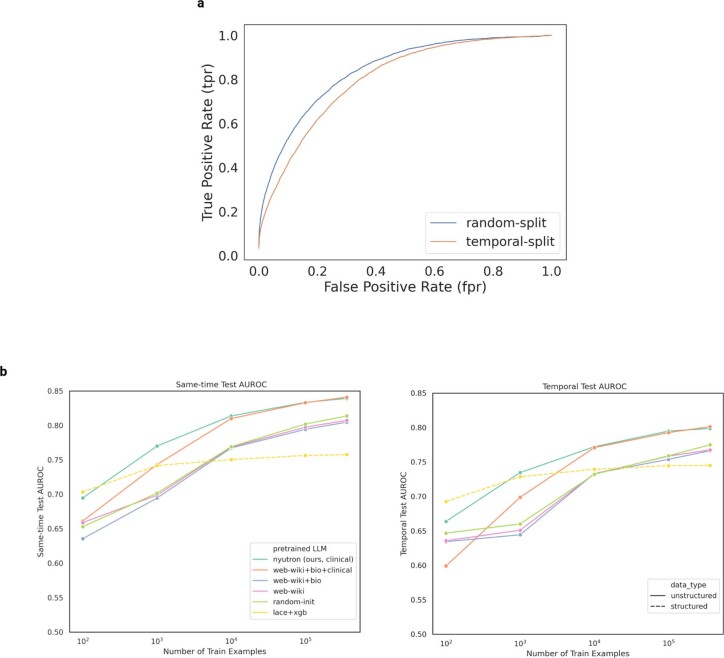
Difference between random test and temporal test. **a**, AUC curve for the random test shows better performance than the temporal test. The random-test AUC is 84.13%, compared to the temporal-test AUC of 80.2%. The difference highlights the importance of creating a test set to reflect the problem setup. In the case of readmission prediction, the deployment set always comes from the future of the training set. Thus we use the temporal test AUC for model selection. **b**, Comparison of random-test AUC and temporal-test AUC as the number of training examples increases shows that temporal-testing is important to estimate deployment performance. Here we show that sampling a temporally split out dataset seems “harder” than a randomly sampled test dataset because all tested LLMs and lace+xgb perform worse on the temporal test (notes from the future) than the random test (notes from the same time as the training data). The colored lines on the left (random test AUCs) are generally higher than the colored lines on the right (temporal test AUCs). We conclude that this is an important distinction that temporally sampled held-out test sets give a more realistic estimate of model performance. Interestingly, the language models seem to be more sensitive to this phenomenon than the lace+xgb model.

The random split does not resemble the deployment scenario, in which the test data come from the future of the training data. We therefore created a temporal split to simulate deployment and observed a meaningful difference in test statistics compared with the random split (the random test AUC was 84.13%, whereas the temporal test AUC was 80.2%), confirming the importance of this second testing phase (further comparison in Extended Data Fig. 1).

**Extended Data Fig. 2:**
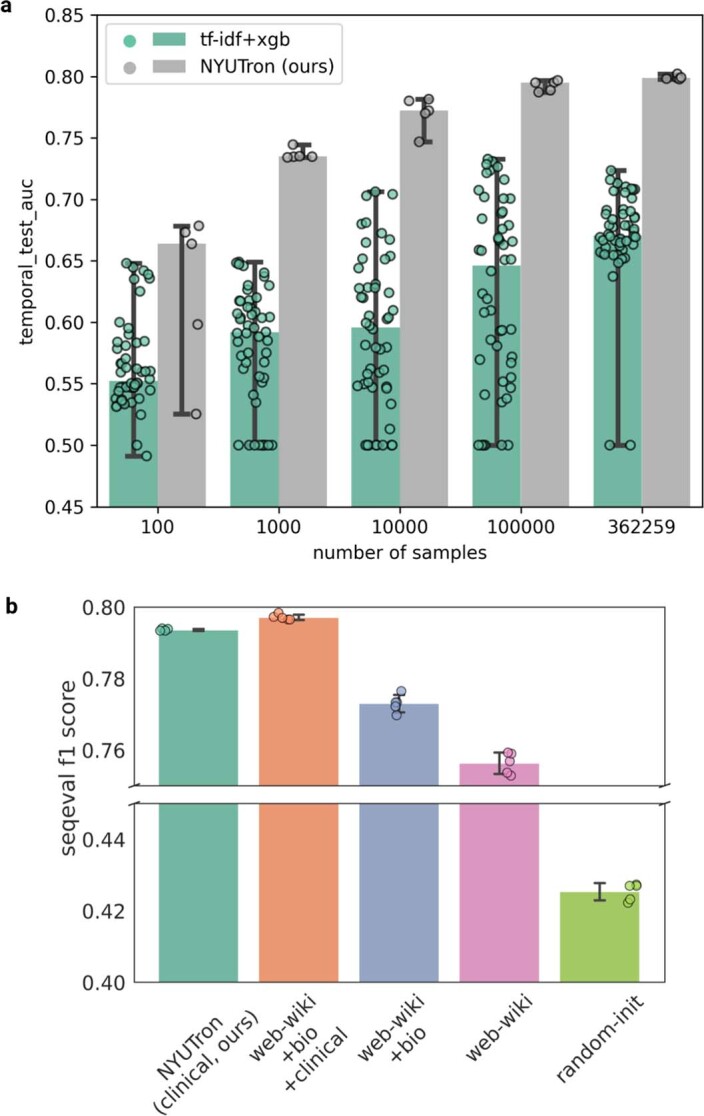
Benchmarking NYUTron against a traditional NLP model and other language models on a different clinical prediction task (clinical concept extraction). We observe a similar trend as readmission prediction: (a) shows that NYUTron has better performance than tf-idf under different data availability settings and (b) shows that clinically pretrained language models have better performance than non-clinically pretrained language models. This corroborates our findings that health-system scale language models are general purpose clinical prediction engines and that a domain match between pretraining and finetuning corpus contributes to task performance. **a**, Comparison of temporal test AUCs between NYUTron and a traditional NLP model (tf-idf+xgb). NYUTron has a higher median AUC than tf-idf+xgb for all tested number of finetuning examples. The black vertical line indicates standard deviation over 5 trials of different random seeds (0, 13, 24, 36, 42). **b**, Comparison of LLMs’ finetuning performances on the NER task. On the i2b2-2012 clinical concept extraction task, the LLMs that are pretrained with clinical corpora (NYUTron, web-wiki+bio+clinical) have a higher average f1 score than LLMs that are not pretrained with clinical corpora (web-wiki+bio, web-wiki, random-init). Specifically, NYUTron and web-wiki+bio+clinical perform better than the randomly initialized model (36.64% higher median seqeval f1 score) and non-clinically pretrained models (2.01%–3.48% higher median seqeval f1 score). Note that the height of each bar is the average f1 score and the half length of each black vertical line indicates the standard deviation over 5 trials of different random seeds (0, 13, 24, 36, 42).

NYUTron is competitive with traditional models and other LLMs. We evaluated the effectiveness of NYUTron by comparing its test performance on the temporal split against that of a traditional model and four different types of LLMs. NYUTron had the highest AUC when fine-tuned with the full dataset (Fig. 3b), with a median AUC of 79.87% ± 0.17%, which was similar to the clinical+web-wiki+bio AUC of 80.14% ± 0.26%. Compared with LLMs pretrained with non-clinical text (web-wiki+bio and web-wiki), NYUTron’s median AUC was 2.37% to 3.23% higher. Compared with the traditional model that uses structured features (lace+xgb), NYUTron had a 5.36% higher AUC. Compared with a model using traditional natural language processing (NLP) embedding (tf-idf+xgb), NYUTron had a 12.8% higher median AUC (Extended Data Fig. 2a).

An LLM trained on unstructured clinical notes better scales with data than traditional structured models. Compared with lace+xgb, NYUTron benefits from an increasing amount of labelled examples and achieved a better AUC when fine-tuned with the full dataset. Figure 3b shows that lace+xgb (dashed yellow line) and NYUTron (solid green line) had similar AUCs at 100 and 1,000 examples. However, NYUTron’s AUC consistently improved with more examples whereas lace+xgb’s AUC started to plateau (from 100 to 1,000 examples, NYUTron’s AUC increased by 7.27% whereas that of lace+xgb increased by 3.98%; from 10,000 to 392,336 examples, NYUTron’s AUC increased by 2.15% whereas that of lace+xgb increased by 0.63%). With the full fine-tuning dataset, NYUTron had a 7.04% higher AUC than lace+xgb.

Pretraining on a large amount of unlabelled clinical notes contributes to performance. Compared with the randomly initialized LLM (random-init), NYUTron learns to generalize better from fewer examples. Figure 3b shows that, whereas NYUTron needed 10,000 examples to achieve an AUC of around 75%, random-init needed 100,000 examples. We also observed a similar trend in another clinical prediction task: NYUTron performed better than the random-init model (36.83% higher F1 score) and the non-clinically pretrained models (2.06% to 3.73% higher F1 score) on the clinical named entity recognition (NER) task from the 2012 i2b2 challenge (Extended Data Fig. 2b).

**Extended Data Fig. 3:**
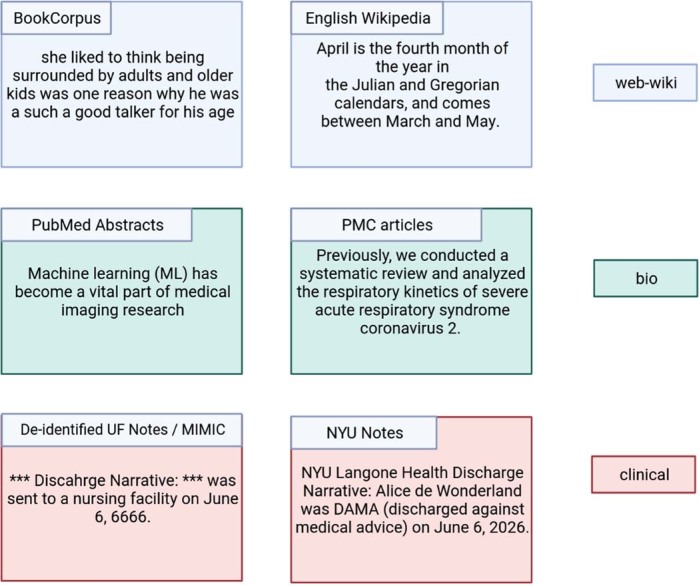
Examples of pretraining corpora. We include here some examples from the utilized pretraining corpora to help contextualize our work. Examples from three types of pretrain corpus: (1) web-wiki (online books from bookcorpus and encyclopedia articles from English Wikipedia), (2) bio (abstracts of academic papers from Pubmed Abstracts and full articles from Pubmed Central), and (3) clinical (NYU Notes, NYU Readmission from Langone EHR and clinical notes from University of Florida Health).

It is beneficial to match the domain of the pretraining corpus and the domain of the fine-tuning corpus. Figure 3b shows three pieces of evidence: LLMs pretrained on non-clinical text (web-wiki and web-wiki+bio) had similar performance as random-init. A separate LLM, web-wiki+bio+clinical, had similar performance as NYUTron. Third, compared with LLMs pretrained on non-clinical text (web-wiki and web-wiki+bio), clinically pretrained LLMs (NYUTron and web-wiki+bio+clinical) learned to generalize better from fewer examples. See Extended Data Fig. 3 for comparison of the pretraining corpus.

Having a close domain match during pretraining is particularly beneficial in the low-data setting during fine-tuning. We compared two language models that were pretrained on clinical text from different hospital systems, NYUTron (NYU Langone Health) and web-wiki+bio+clinical (University of Florida). Figure 3b shows that, at 1,000 examples, NYUTron (the in-domain model) had a higher AUC for NYU Langone readmission prediction than web-wiki+bio+clinical (the out-of-domain model). Notably, NYUTron’s advantage disappeared as the number of fine-tuning examples increased, suggesting that sufficient in-domain fine-tuning can adapt models that were pretrained out of domain.

**Extended Data Fig. 4:**
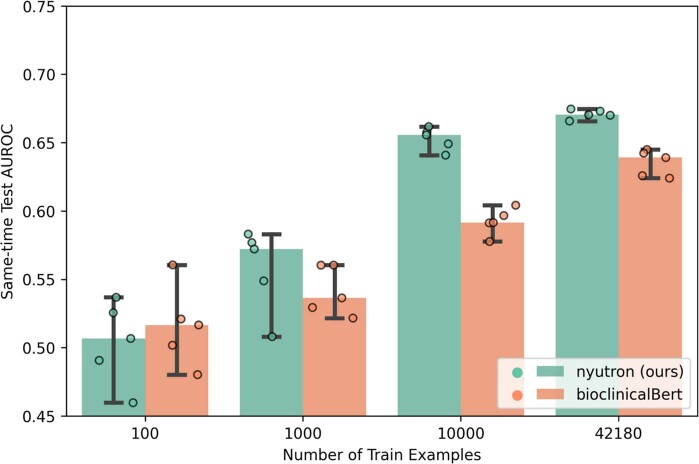
Comparison of NYUTron’s and BioClinicalBERT’s performance on MIMIC-III Readmission. To test how much finetuning NYUTron needs to generalize to another health system, we finetune NYUTron and BioClinicalBERT (which has the same number of parameters and architecture as NYUTron, but pretrained on MIMIC notes, bookcorpus, pubmed and wikipedia articles) using different subsamples of MIMIC-III readmission dataset. The dataset contains 52,726 de-identified ICU discharge notes from Boston Beth Israel Hospital with 8:1:1 train-val-test split. At 100 samples, the AUC is similar. At 1000 samples, NYUTron has a 3.58% higher median AUC than BioClinicalBERT (57.22% v.s. 53.64%). At 10,000 samples, NYUTron has a 6.42% higher median AUC than BioClinicalBERT (65.56% v.s. 59.14%). Using the full dataset (42,180 samples), NYUTron has a 3.8% higher median AUC than BioClinicalBERT (67.04% v.s. 63.24%). Given that NYUTron was pretrained on identified all-department notes from NYU Langone and finetuned on de-identified ICU-specific notes from Beth-Israel, this result shows that NYUTron is able to generalize to a very different health environment through local finetuning. The height of the bar indicates the median performance of 5 experiments using distinct random seeds (0, 13, 24, 36, 42) and the error bar indicates the min-max range.

**Extended Data Fig. 5:**
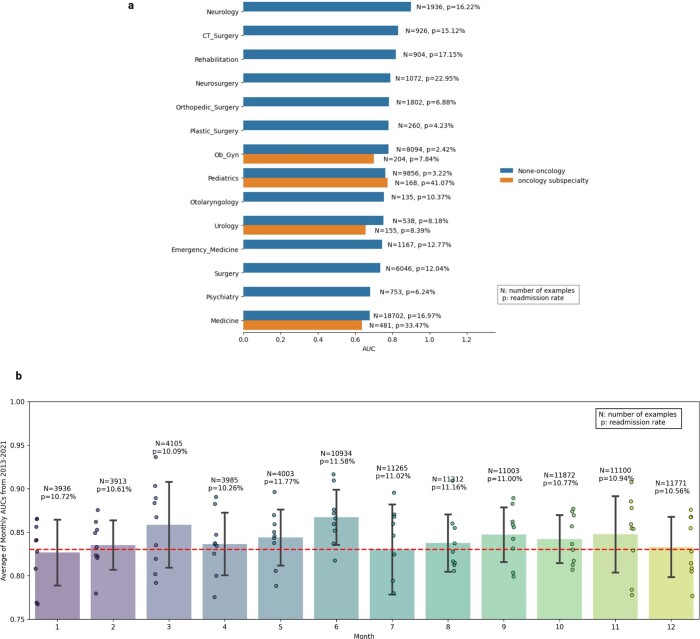
Bias analysis stratifying NYUTron’s performance by clinical departments and months. **a**, A stratified analysis of NYUTron’s temporal test performance by clinical department and oncological subspecialty. NYUTron performs best in the Neurology Department (AUC 90.12%), and performs worst in the Internal Medicine Department (AUC 67.95% for non-oncology specialty and AUC 63.77% for oncology specialty), with a difference of about 20% AUC. This significant variance across clinical departments suggests that a more fine-grained analysis may lead to performance benefits. We annotate the number of examples (N) and the readmission rate (p) for each department. **b**, NYUTron’s performance displays minor fluctuations over months. We plot the average monthly test AUC of NYUTron from January 2013 to December 2021 to look for underlying monthly trends or cycles and to test the hypothesis that performance would be worst in July when new physicians start their training with a different writing style than physicians already in practice (dashed red line indicating the monthly AUC of July). The height of the bar indicates average monthly performance across the 9 years and the vertical bar indicates the standard deviation. We annotate the number of examples (N) and the readmission rate (p) for each month. We note that July has the second lowest monthly AUC and the highest variance. We speculate (and need more years of data to verify) that clinical notes written by new physicians are associated with the temporal shift across the months and the drop in performance in July. Average AUCs from the quarters January to March, April to June, and July to September are increasing, which may coincide with residents’ rotation schedule across different clinical departments. We leave further investigation of this cyclical performance to future work.

**Extended Data Fig. 6:**
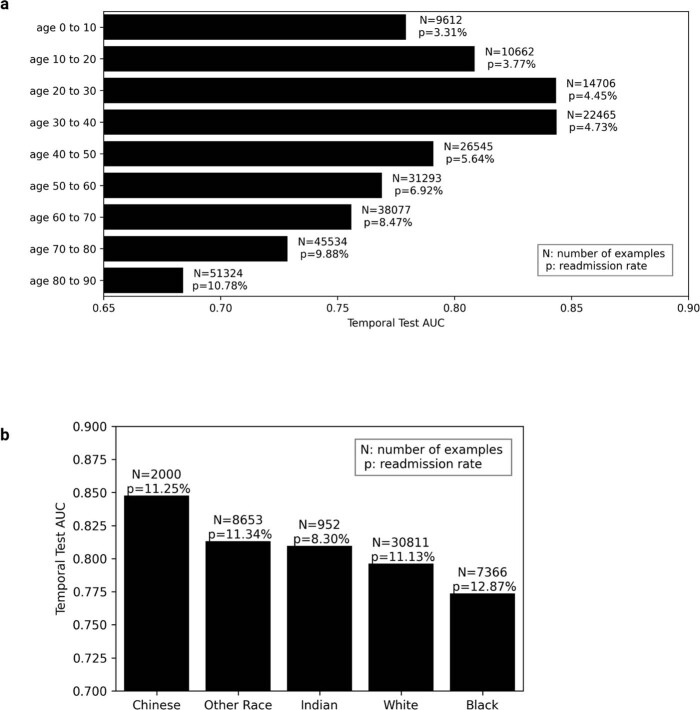
Bias analysis stratifying NYUTron’s performance by age groups and major racial groups. As part of an analysis of model performance by two possible sources of bias, age and race, we perform stratified analyses of NYUTron’s performance. We annotate the number of examples (N) and the readmission rate (p) for each evaluation. **a**, We stratify the temporal test based on nine bins of ages (0 to 90 years with bins of 10 year intervals). NYUTron performs best for patients who are 10 to 40 years old, and has declining performance by decile over the age of 40 years with the worst performance in the 80–90 years of age group. We observe that this isn’t an effect of sample size, the single largest sample is age 80–90, but likely reflects complexity and comorbidity burdens being disproportionately higher with advanced age. **b**, To test for potential dependencies and bias by race, we first identify the five most frequent races in the dataset (White, Other Race, Black, Chinese, Indian), then stratify the evaluation results by race. NYUTron performs best on Chinese patients and worst on Black patients with a mild variation in AUC across both groups.

Clinical language models show generalizability to different sites through local fine-tuning. To investigate the robustness of NYUTron across clinical environments, we chose two hospitals that are geographically separated within the NYU Langone Health System. For brevity, we refer to Tisch Hospital in Manhattan as ‘Manhattan’, NYU Langone Hospital–Brooklyn as ‘Brooklyn’ and all four hospitals within the NYU Langone Health System (Manhattan, Brooklyn, NYU Langone Orthopedic Hospital and NYU Langone Hospital–Long Island) as ‘all sites’. We considered three LLMs pretrained on different sites: the first one was pretrained in Manhattan, the second one was pretrained in Brooklyn and the third one was pretrained on all sites. For each of the pretrained LLMs, we fine-tuned the LLM with a readmission dataset from either Manhattan or Brooklyn. Finally, we asked the fine-tuned LLM to predict readmission on the basis of discharge notes from either Manhattan or Brooklyn. Figure 3c,d shows that the LLM pretrained on all sites had the best performance on both ‘test Manhattan’ and ‘test Brooklyn’. For all the LLMs, fine-tuning with the local dataset (‘fine-tune Manhattan/Brooklyn’) led to a higher test AUC at the test site (‘test Manhattan/Brooklyn’) compared with fine-tuning at another site (‘fine-tune Brooklyn/Manhattan’). Therefore, pretraining with data from all sites and local fine-tuning is the best way to optimize performance. We performed additional analyses that showed that NYUTron is able to generalize to a different health system through local fine-tuning (Supplementary Information section 4.1 and Extended Data Fig. 4) and compared the robustness of NYUTron and lace+xgb with respect to training sites (Supplementary Information section 4.2). We also found that NYUTron is sensitive to notes from different clinical departments and patients with different demographics and that its performance fluctuates over months (Extended Data Figs. 5 and 6). The causes of the discrepancies can be very complex (discussed in Supplementary Information section 4.3) and will be studied in future work.

## Prospective study of readmission

**Fig. 4:**
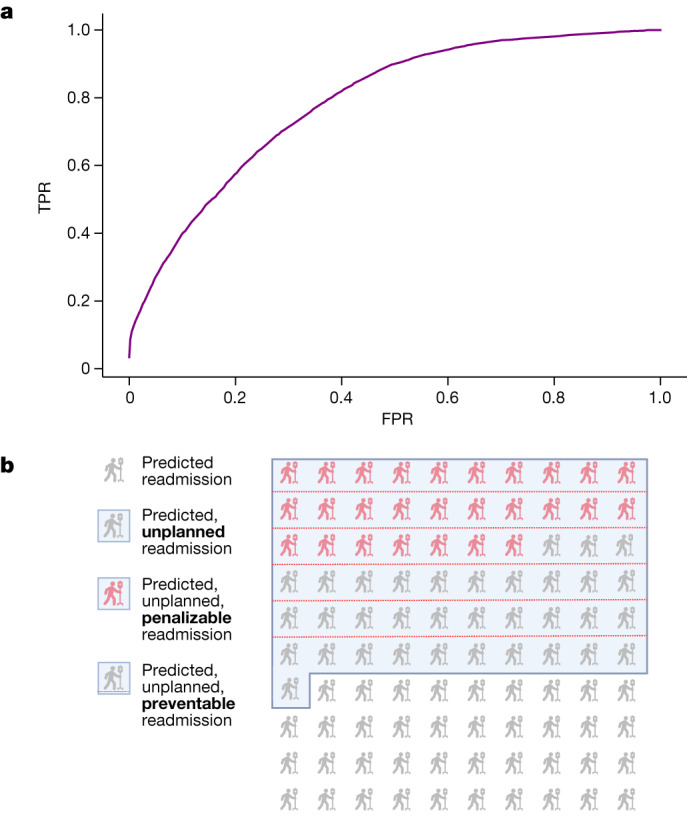
Prospective study of NYUTron’s predictive performance. **a**, NYUTron had an AUC of 78.70% in a prospective, single-arm, non-interventional trial with recall of 82.3% and precision of 20.6%. **b**, A panel of six physicians reviewed NYUTron’s results for potential clinical impact. Of 100 readmissions that were successfully identified by NYUTron, 61% were unplanned readmissions, 50% would have resulted in a penalty under CMS guidelines and 27% were preventable at the time of discharge according to the consensus opinion of the multi-specialty panel of physicians who reviewed cases from the prospective trial. See Supplementary Information section 2.1 for a discussion of the readmission label and the practical significance of the observed performance.

To assess NYUTron’s performance outside the development environment, we selected a model on the basis of the retrospective trial results and ran a prospective trial from January to April 2022. During this time period, we deployed NYUTron in an accelerated format and loaded it into an inference engine, which interfaces with the EHR, to read discharge notes as they were signed by treating physicians. In this period, there were 29,286 discharged encounters, with 3,271 patients (11.17%) returning within 30 days. NYUTron predicted 2,692 of the 3,271 readmissions (82.30% recall) with 20.58% precision. Figure 4a shows that NYUTron had an AUC of 78.70%.

To gauge the potential clinical impact, a group of six physicians performed a qualitative evaluation of 100 randomly sampled readmitted cases that were captured by NYUTron following the trial’s conclusion. Physician review suggested that some true positive predictions by NYUTron are clinically meaningful, preventable readmissions. Overall, readmitted patients who were predicted to be readmitted were 6.02 times more likely to die in hospital and stay 2.93 days longer (*P* < 10^−4^). As shown in Fig. 4b, 61% of the predicted case were unplanned, and the mean predicted probabilities for these unplanned readmissions were lower than those for planned readmissions (31.9% ± 31.1% versus 82.1% ± 27.3%; *P* < 10^−4^). Among the unplanned readmissions, 19.67% of patients experienced an adverse event or death on readmission, with 50% of these events considered preventable by the physician panel. From a financial standpoint, 81.9% of the unplanned readmissions would be penalized according to Centers for Medicare and Medicaid Services (CMS) guidelines. Among the penalizable cases, 54% were considered preventable. Notably, 3 of the 27 preventable readmissions had *Clostridioides difficile* enterocolitis, a contagious, healthcare-associated bacterial infection that causes 1 in 11 people over age 65 to die within 1 month^21^.

## Discussion

We present our work in developing, training, validating and deploying NYUTron, a health system-scale LLM designed and validated for clinical use. We demonstrate NYUTron’s performance on three clinical tasks (in-patient mortality prediction, comorbidity index prediction and readmission prediction) and two operational tasks (insurance claim denial prediction and inpatient LOS prediction). We also performed a detailed analysis of readmission prediction owing to its clinical and operational importance and its well-documented history in the medical informatics literature. We view the flexibility of our approach in using an encoder architecture (BERT), which relies on only unstructured text inputs to generate a single prediction, as being a virtue, and we anticipate many future tasks built on this fundamental paradigm to assist with multiple aspects of patient care and automating hospital operations.

An ethical consideration in deployment is that physicians and administrators could over-rely on NYUTron’s predictions owing to its seamless integration with existing medical workflows, thereby leading to undesirable outcomes. Further research is needed to optimize human–AI interactions, as well as development of standardized assessments for sources of bias or other unexpected failure points. Ongoing work from our group around measuring the similarity between language models’ sensitivity patterns and those of physicians through token-level perturbations of the clinical notes^22^ is one among many such efforts.

Large, generative LLMs also present a unique opportunity for integration into medical workflows; however, they are highly dependent on user inputs and prompting^23^ and are not as easily adapted for automation of basic clinical and operational tasks. The seamless integration into existing medical informatics workflows is a virtue of our approach, and we hope that this work presents itself as a flexible solution to the last-mile problem—any structured data algorithm can be reconceptualized and rapidly prototyped within this framework. As part of monitoring the impact of such a system on physician behaviour and on patients, there should be a level of continuous supervision to capture human–machine interactions, as well as mitigate the risk of model drift over time. We discuss our implementation of such a system in Supplementary Information section 5.

Our approach of using a smaller (<1 billion parameters) encoder language model trained on highly tailored data represents a marked departure from the current trend in language model research that focuses on massive (>1 billion parameters), generative models pretrained on large, non-specific datasets. Nonetheless, even relatively small LLMs, such as the ones used in this study, require a substantial amount of compute time for pretraining. Our pretraining used 24 NVIDIA A100 GPUs with 40 GB of VRAM for 3 weeks, and our fine-tuning used 8 A100 GPUs for 6 hours per run. This amount of computation is not commonly accessible to research groups, although we note that it is less than that in similar LLM projects routinely pursued by industry research groups and that our results indicate that massive pretraining may not be necessary to obtain highly performant models. Our results show that high-quality datasets for fine-tuning are more valuable than pretraining, and, on the basis of our experimental results, we recommend that users locally fine-tune an externally pretrained language model when computational ability is limited. Regarding the choice for the externally pretrained model, we further recommend using a model pretrained with a large amount of in-domain clinical text, although we note that large, out-of-domain models can be highly performant, particularly when combined with in-domain fine-tuning. Work with larger decoder-based architectures has also demonstrated a benefit with fine-tuning on medical data or prompt tuning with chain of thought, instructions and related techniques^24,25^, which further emphasizes the necessity of accounting for the domain shift from general to medical text for LLM work in the medical sciences. Although we have not compared these approaches directly (which would require more medical text or fusion with general-domain text for training a compute-optimal model^26^), we believe that this could be an interesting future direction for research and that, in the end, approaches combining these different approaches to language modelling may prove to be complementary depending on the use case.

The ultimate validation of our approach must come from randomized controlled trials of interventions tied to individual task predictions to assess their clinical impact and from user feedback as we continue to integrate NYUTron into health systems. As we plan this within our own health system, we recommend the consideration of different levels of intervention depending on the predicted risk of patients for each task. For instance, for a patient at low risk for 30-day readmission, follow-up calls could be scheduled; for a high-risk patient, care should be taken to limit premature discharge. All interventions should be decided on with physician supervision, although many of the operational uses can probably be fully automated.

It is a long-standing dream for physicians to have AI assistants observing care along with them and chiming in with predictions and advice. To take a step towards this futuristic vision, we trained an LLM, NYUTron, on the entire EHR of a large healthcare system to read physician notes and make several of these predictions across a wide range of clinical and operational tasks. We deployed NYUTron in a live healthcare environment and demonstrate its efficacy at predicting 30-day readmission while being integrated seamlessly into clinical workflows. We believe that this work opens the door to translating the progress in modern natural language processing and deep learning to improving the quality and affordability of healthcare, and we are excited to see what comes next.

## Methods

### Pretraining datasets

#### NYU Notes

We created this dataset of unlabelled clinical notes directly from the NYU Langone EHR. The dataset contains 387,144 patients, 7,247,694 notes and 4,112,249,482 words in total. We built NYU Notes as follows: we wrote structured query language (SQL) scripts to query the NYU Langone EHR. We first prototyped the queries with an interactive web-based editor (Cloudera Hue) and then download the query results as comma-separated files (CSVs) to NYU Langone’s high-performance computing cluster. We included notes signed by medical professionals (physicians, residents, physician assistants, nurse practitioners and fellows) at Tisch Hospital, NYU Langone Hospital–Brooklyn, NYU Langone Hospital–Long Island and NYU Langone Orthopedic Hospital from 2011 to 2020 (inclusive). We excluded any notes that were derived from billing, labelled as invalid or empty. We split the notes into three sets, training, validation and test sets, with a ratio of 949:50:1. Lastly, we masked tokens with 15% probability to create masked text and labels.

#### NYU Notes–Manhattan

We created this dataset of unlabelled clinical notes as the subset of NYU Notes that were written in Tisch Hospital in Manhattan. The dataset contains 256,217 patients, 4,342,602 notes and 2,381,466,993 words in total.

#### NYU Notes–Brooklyn

We created this dataset of unlabelled clinical notes as the subset of NYU Notes that were written in NYU Langone Health–Brooklyn. The dataset contains 104,521 patients, 1,337,352 notes and 1,102,078,012 words in total.

### Fine-tuning datasets

#### NYU Readmission

**Extended Data Fig. 8:**
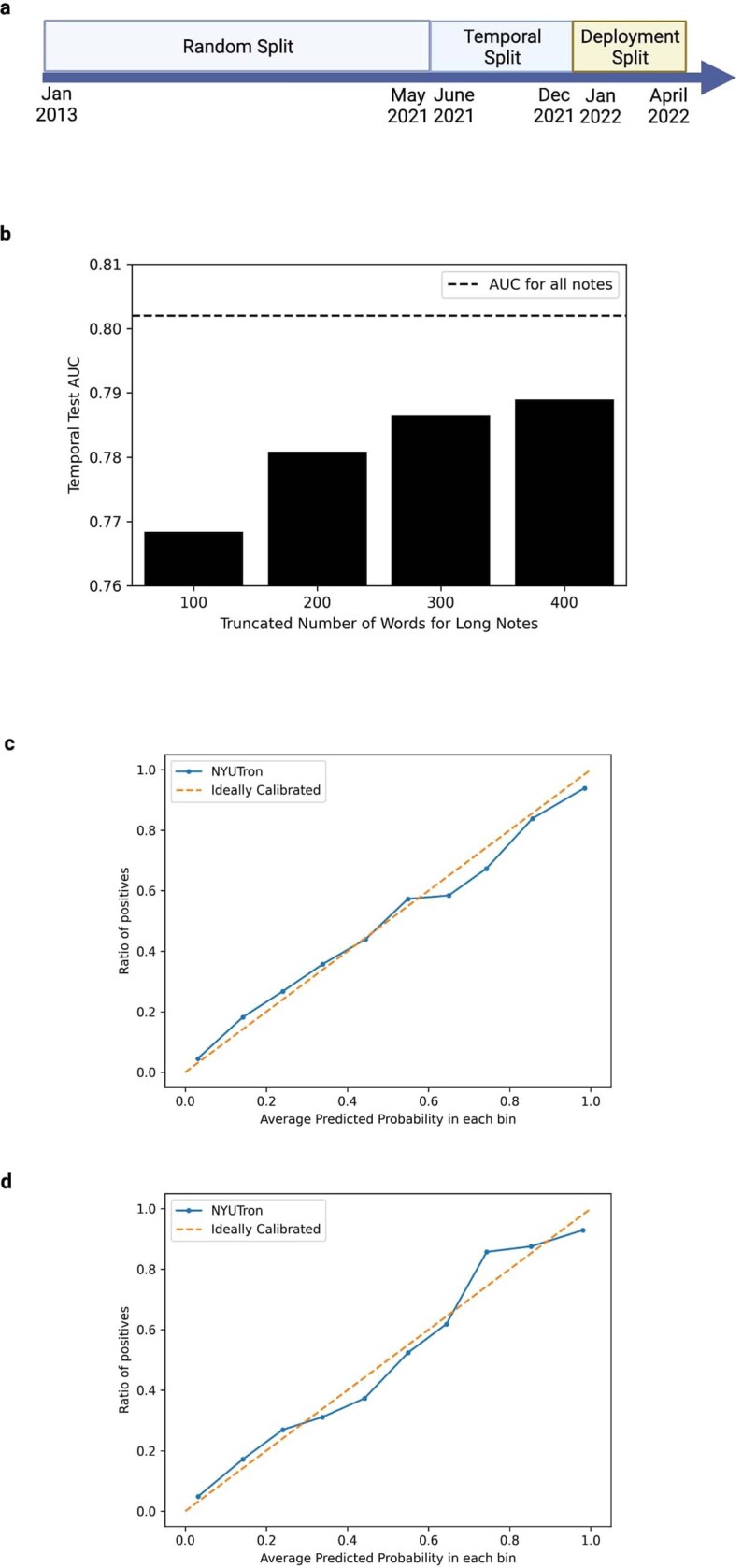
Additional information about readmission prediction. **a**, Visualization of readmission data split timelines. We visualize the random split, temporal split, and deployment split on a timeline to indicate this decision for model evaluation. The random split starts from January 2013 and ends in May 2021 (inclusive), which is further split into a 80% train set, 10% validation set and a 10% test set. The temporal split (temporal test) starts from June 2021 and ends in December 2021, a time period from which no training samples were sampled from. The deployment data is necessarily sampled from the future as it is accrued prospectively as part of our single arm, non-interventional clinical trial. **b**, NYUTron’s performance increases with more complete input notes. To attempt to estimate performance as a function of sequence length we sampled a subset of “long notes” from the temporal test set. Each note in this subset has no less than 400 words, or approximately 512 tokens. We truncated these long notes to 100, 200, 300 and 400 words while keeping their readmission labels fixed in order to demonstrate the incremental gain in performance as we capture proportionally more information from each of these “long notes”. The dashed line is the AUC of all notes. This figure shows that processing more words from the possible input leads to a better evaluation performance and confirms that there is a clear potential for improving performance by increasing maximum sequence length. **c**,**d** NYUTron’s calibration curve for temporal test (c, number of evaluation examples is N = 53,916) and prospective deployment (d, number of evaluation examples is N = 29,286). As a reference, the orange line is the calibration curve of an ideally calibrated classifier. The blue line is NYUTron’s calibration curve. Currently we do not perform any additional calibration and choose the decision threshold based on the precision and recall on the temporal validation set. The predicted probability is normalized by the largest predicted probability. Overall the model is well calibrated to the 30-day readmission task.

We created this dataset of labelled discharge notes (with binary labels for readmission) from the NYU Langone EHR. Most of the notes from this dataset are a subset of NYU Notes, with additional discharge notes from 2021 for the temporal test. The dataset contains 413,845 patients, 506,740 notes and 487,395,462 words in total. We built this dataset as follows: for each encounter that ended between January 2011 and November 2021, we included its discharge note with a binary label for 30-day all-cause readmission. We assigned the ‘readmitted’ label if the patient had an admission note within 30 days of being discharged. To focus on modelling acute care readmission, we excluded discharge notes from the rehabilitation, dialysis and palliative care departments because these were not acute care admissions. We split the dataset into four sets: training, validation, test and temporal test sets. The first three sets were notes from January 2011 to May 2021, with a ratio of 8:1:1. The temporal test set included notes from June to December 2021. See Extended Data Fig. 8a for a visualization of the four-way split.

#### NYU Readmission–Manhattan

We created this dataset of unlabelled clinical notes as the subset of notes in the NYU Readmission dataset that were written in Tisch Hospital in Manhattan. The dataset contains 240,824 patients, 296,519 notes and 253,622,053 words.

#### NYU Readmission–Brooklyn

We created this dataset of unlabelled clinical notes as the subset of clinical notes from the NYU Readmission dataset that were written in NYU Langone Health–Brooklyn. The dataset contains 94,653 patients, 113,275 notes and 142,767,957 words.

#### NYU Mortality

We created this dataset of history and physical (H&P) notes with binary labels for in-hospital mortality from the NYU Langone EHR. Most of the notes from this dataset are a subset of NYU Notes, with additional H&P notes from 2021 for the temporal test. The dataset contains 371,922 patients, 469,162 notes and 484,467,141 words in total. We built this dataset as follows: for each encounter that ended between January 2011 and November 2021, we included its H&P note with a binary label for in-hospital mortality. We assigned the positive label if the patient’s discharge disposition was ‘expired’. We split the dataset into four sets: training, validation, test and temporal test sets. The first three sets were notes from January 2011 to May 2021, with a ratio of 8:1:1, and the temporal test set included notes from June to December 2021.

#### NYU Binned Comorbidity

We created this dataset of H&P notes with five class labels for hospital LOS from the NYU Langone EHR. Most of the notes from this dataset were a subset of NYU Notes, with additional H&P notes from 2021 for the temporal test. The dataset contains 327,039 patients, 403,579 notes and 422,485,417 words in total. The dataset contains fewer labelled encounters than the NYU Mortality and NYU Binned LOS datasets because 22% of the encounters had no International Classification of Diseases (ICD) codes to calculate the CCI score. This missingness motivated our task of predicting binned CCI score with a lack of structured ICD codes. We built this dataset as follows: for each encounter that ended between January 2011 and November 2021, we included its H&P note with a five-class label for binned CCI score. To generate the labels, we first calculated the comorbidity index using the ICD codes and the scoring function in ref. ^27^. We then discretized the scores into five classes: we assigned label 0 for a comorbidity index below the 50% quantile (0 days), label 1 for a comorbidity index between the 50% and 75% quantile (1–2 days), label 2 for a comorbidity index between the 75% and 90% quantile (3–4 days), label 3 for a comorbidity index between the 90% and 99% quantile (4–7 days) and label 4 for a comorbidity index above the 99% quantile (>7 days). We split the dataset into four sets: training, validation, test and temporal test sets. The first three sets were notes from January 2011 to May 2021, with a ratio of 8:1:1, and the temporal test set included notes from June to December 2021.

#### NYU Binned LOS

We created this dataset of H&P notes with quantile labels for hospital LOS from the NYU Langone EHR. Most of the notes from this dataset were a subset of NYU Notes, with additional H&P notes from 2021 for the temporal test. The dataset contains 371,922 patients, 469,162 notes and 484,467,141 words in total. We built this dataset as follows: for each encounter that ended between January 2011 and November 2021, we included its H&P note with a binary label and a quantile label for LOS. For the quantile label, we assigned label 0 for an LOS below the 25% quantile (0–2 days), label 1 for an LOS between the 25% and 50% quantile (3 days), label 2 for an LOS between the 50% and 75% quantile (4–5 days) and label 3 for an LOS above the 75% quantile (>5 days). We split the dataset into four sets: training, validation, test and temporal test sets. The first three sets were notes from January 2011 to May 2021, with a ratio of 8:1:1, and the temporal test set included notes from June to December 2021.

#### NYU Insurance Denial

We created this dataset of H&P notes with binary labels for whether the patient’s insurance claim was initially rejected or directly approved. The dataset contains 54,563 patients, 55,791 notes and 51,270,256 words in total. We built this dataset as follows: for each encounter that occurred between May 1, 2021, and April 30, 2022, we included its H&P note with a binary label for insurance denial. We assigned a positive label if the patient’s insurance claim status was ‘final, adverse determination’ (claim was rejected by insurance and was again rejected following appeal) or ‘final, favorable determination’ (claim was rejected by insurance and approved following appeal). We split the dataset into four sets: training, validation, test and temporal test sets. The first three sets were notes from May 1, 2021, to February 30, 2022, with a ratio of 18:1:1. The temporal test set included notes from March 1 to April 30, 2022.

#### NYU Insurance Denial–Discharge Notes

We created this dataset of discharge notes with binary labels for whether the patient’s insurance claim was initially rejected or directly approved. The dataset contains 54,563 patients, 55,791 notes and 49,405,133 words in total. We built this dataset as follows: for each encounter that occurred between May 1, 2021, and April 30, 2022, we included its discharge note with a binary label for insurance denial. The label assignment and four-way split were the same as in the NYU Insurance Denial dataset.

#### NYU Insurance Eventual Denial, H&P

This dataset contained the same notes as the NYU Insurance Denial dataset, but the labels were different. The binary label indicated whether the patient’s insurance claim was eventually rejected (even after appeal) or was eventually approved (direct approval or approval after appeal).

#### NYU Insurance Eventual Denial–Discharge

This dataset contained the same notes as the NYU Insurance Denial–Discharge Notes dataset, but the labels were different. The binary label indicated whether the patient’s insurance claim was eventually rejected (even after appeal) or was eventually approved (direct approval or approval after appeal).

#### i2b2-2012 NER

This is an open dataset released by the Harvard Medical School as part of an annual clinical NLP challenge^28^. This dataset is a well-known benchmark in the clinical NLP community. The task is to identify and classify clinical concepts (for example, treatments), clinical departments (for example, surgery), occurrences of events (for example, admission) and evidentials (for example, the patient complained) from de-identified clinical notes from Beth Israel Medical Center in Boston. The dataset contains no more than 310 patients, 310 notes and 636,000 words. We downloaded the dataset as a compressed tar.gz file from the n2c2 data portal after our use application was approved.

#### MIMIC-III Readmission

This is an open dataset for an intensive care unit (ICU) EHR released by MIT and Boston Beth Israel Medical Center^29^. We collected a set of 52,726 discharge notes and created a 30-day all-cause readmission label by checking whether there was any subsequent encounter within 30 days. The readmission rate was 6%. We split the data into training, validation and test sets in a 8:1:1 ratio.

### Deployment dataset

#### NYU Readmission–Deployment

This dataset consists of discharge notes with binary labels for readmission from our deployment engine and the NYU Langone EHR. From January to April 2022, every time a discharge note was signed by a physician, the note was sent to our custom inference engine for NYUTron’s prediction. The paired discharge note and prediction were recorded in a database. The database contained 27,376 patients, 29,287 notes and 34,669,963 words by the end of the study period.

### Structured datasets

#### NYU Readmission–LACE

We created this dataset of structured LACE^30^ features with binary labels for readmission for comparison against the unstructured models. The dataset contains structured features for all encounters in the NYU readmission dataset. LACE is a traditional clinical prediction rule for readmission with four features: LOS, acuity of readmission, Charlson comorbidity index, and number of recent emergency department visits in the past 6 months. We built the dataset as follows: for every encounter in the NYU Readmission dataset, we collected data on the four LACE features from the NYU Langone EHR. LOS was the difference (in days) between the discharge date and the admission date. Acuity of readmission was a binary feature indicating whether the patient was admitted to the emergency department. The comorbidity index was calculated with the ICD-9 or ICD-10 codes for chronic diseases, on the basis of the mapping algorithm in ref. ^31^ and the scoring function in ref. ^27^. The number of emergency department visits was calculated from the patient’s encounter history up to 6 months before the admission date.

#### NYU Readmission–LACE, Manhattan

We created this dataset of structured LACE features from the subset of notes from the NYU Readmission–LACE dataset that were written in Tisch Hospital in Manhattan.

#### NYU Readmission–LACE, Brooklyn

We created this dataset of structured LACE features from the subset of notes from the NYU Readmission–LACE dataset that were written in NYU Langone Health–Brooklyn.

#### NYU Mortality–SAPS2 + APACHE2

We created this dataset of structured SAPS2 + APACHE2 features with binary labels for in-hospital mortality to compare against the unstructured data. The dataset contains a subset of structured SAPS2 + APACHE2 features for all encounters in the NYU Mortality dataset. SAPS2 + APACHE2 features are a subset of the features used in the SAPS2 model^15^ and the APACHE2 model^16^ for ICU mortality prediction. We selected the subset of features that were available in the NYU Langone EHR. We included the following 12 features: age (numerical), mean heart rate (numerical), systolic blood pressure (numerical), atrial temperature (numerical), blood urea nitrogen concentration (numerical), sodium concentration (numerical), potassium concentration (numerical), bilirubin concentration (numerical), white blood cell count (numerical), pH (numerical), creatine concentration (numerical) and haematocrit (numerical). We additionally included department specialty (categorical). We excluded the following features owing to their unavailability: PaO_2_/FiO_2_ (ratio of arterial oxygen partial pressure to fractional inspired oxygen), whether the patient was on mechanical ventilation or continuous positive airway pressure (CPAP), bicarbonate concentration, urine output, Glasgow Coma Scale score, presence of metastatic cancer or haematological malignancy or AIDS, and whether the admission was scheduled.

#### NYU Binned LOS–Lisbon Portugal

We created this dataset of structured ‘Lisbon Portugal’ features with binary labels for in-hospital mortality to compare against the unstructured data model. The dataset contains a subset of the features used in the Lisbon Portugal dataset^18^ (which is widely used in the LOS prediction literature) for all encounters in the NYU Binned LOS dataset. We selected a subset of 12 features that were available in the NYU Langone EHR: gender (categorical), age as measured by the difference in years between the birth date and the admission date (numerical), highest level of education (categorical), country (categorical), postal code as address (categorical), marital status (categorical), admission type (categorical), admission service type (categorical), provider ID (categorical), department specialty (categorical), procedure name (categorical) and number of previous admissions (numerical). We left out diagnosis because it is not always available at the time of writing H&P notes. We excluded the following three features owing to difficulty in finding them in the NYU Langone EHR: homogeneous group diagnosis code, great diagnostic category and treatment.

#### NYU Insurance Denial–Claim Forms

We created this structured dataset based on the NYU Insurance Denial dataset for comparison against the unstructured data model. The dataset contains structured features for all encounters in the NYU Insurance Denial dataset and has the same splits as the NYU Insurance Denial dataset. Selection of structured features was based on the features in ref. ^19^, which built a model that predicts insurance claim denial from demographic and care-related features found in the claim form. We found eight available features in the NYU Langone EHR: patient name (categorical), age (numerical), gender (categorical), postal code as a generalization of address (categorical), insurance brand (categorical), first insurance plan name (categorical), provider ID (categorical) and provider type (categorical). We additionally added four features based on the clinician’s inputs: second insurance plan code (categorical), a binary flag for surgical cases (categorical), a binary flag for emergency department cases (categorical) and a binary flag for Medicare fee-for-service users (categorical). We left out six features in ref. ^19^ owing to difficulty in searching for them: the patient’s relationship to the insured person, network type, whether the claim was a resubmission, diagnosis pointer, charge of service and prior authorization number.

### Preprocessing

#### Pretraining datasets (NYU Notes, NYU Notes–Manhattan, NYU Notes–Brooklyn)

Using these datasets, we trained an uncased BERT wordpiece tokenizer with a vocabulary size of 50,000 tokens, a maximum sequence length of 512 tokens and special tokens [SEP], [PAD], [UNK], [MASK] and [CLS]. Because most of the clinical notes had more than 512 tokens, we split each long note into non-overlapping chunks that were under the maximum sequence length. Specifically, we split each note into sentences using natural language toolkit (nltk)^32^ and tokenized each sentence. For sentences that were longer than 512 tokens, we truncated them. Next, for all tokenized sentences in the same note, we concatenated them into groups such that each group had exactly the maximum sequence length. We discarded any remaining group (with a length strictly less than the maximum) of a long note.

#### Fine-tuning datasets (NYU Readmission, NYU Readmission–Manhattan, NYU Readmission–Brooklyn, NYU Mortality, NYU Binned LOS, NYU Insurance Denial, NYU Binned Comorbidity)

Using the tokenizer trained with NYU Notes, we first tokenized the discharge note. We truncated notes that exceeded the maximum sequence length of 512 tokens. We leave it for the future to design a language model that efficiently reads longer clinical notes (see Extended Data Fig. 8b for the impact of note length on language model performance).

#### i2b2-2012 NER

We first decompressed the tar.gz files into folders of xml files. We then converted the xml files to brat format. Next, we converted the brat files to bio files. Finally, we wrote a custom HuggingFace^33^ data loader to convert the folder of bio files into a HuggingFace dataset. Our code for preprocessing is available at GitHub.

#### Deployment datasets

We first cleaned the notes by stripping out html artifacts. We then tokenized the discharge note using NYUTron’s tokenizer. We truncated notes that exceeded the maximum sequence length of 512 tokens.

#### Structured dataset (NYU Readmission–LACE, NYU Mortality–SAPS2 + APACHE2, NYU Binned LOS–Lisbon Portugal, NYU Insurance Denial–Claim Forms)

When there was a missing numerical feature (for example, the average heart rate was NaN), we filled in the feature as the average feature across the training set. For missing categorical features (for example, the admitting department was ‘unspecified’), we left them as category ‘none’.

### Pretraining

We pretrained a 109 million-parameter BERT model using preprocessed NYU Notes and the MLM objective for 3 weeks (96 epochs) on 24 NVIDIA A100 GPUs distributed over three compute nodes until the validation loss started to plateau. The model has 12 hidden layers with dimension 768, with 12 attention heads per layer. We used a per-device training batch size of 64 and saved every 2,000 steps. We used the Zero Redundancy AdamW optimizer (an improvement over the Adam optimizer) with a constant learning rate of 5 × 10^−5^, FP16 mixed precision and stage 2 parallelization^34–36^.

### Fine-tuning

#### NYUTron + discharge notes for readmission prediction

We replaced the trained MLM classifier with a randomly initialized linear classifier after the last hidden layer of the pretrained BERT model. We fine-tuned the model end to end using the training set of the NYU Readmission dataset for ten epochs, evaluating the validation AUC every half epoch and stopping early with a patience of five. We used the following hyperparameters from manual tuning based on the validation AUC: a learning rate of 2 × 10^−5^, a weight decay of 0.01 and a per-device batch size of 4. We optimized the cross-entropy loss using the AdamW optimizer. While varying the size of the dataset (*N* ∈ {10^2^, 10^3^, 10^4^, 10^5^, 3.92336 × 10^5^}), we fine-tuned the pretrained model using subsamples of the NYU Readmission dataset and evaluated their AUC on the temporal test set. For each size of subsample, we ran five experiments with distinct random seeds (0, 13, 24, 36, 42). For comparison, we looked at the median AUC and the standard deviation of the five experiments.

#### NYUTron + H&P notes for in-hospital mortality prediction

We replaced the trained MLM classifier with a randomly initialized linear classifier after the last hidden layer of the pretrained BERT model. We fine-tuned the model end to end using the training set of the NYU Mortality dataset for ten epochs, evaluating the validation AUC every half epoch and stopping early with a patience of 5. We used the following hyperparameters from manual tuning based on the validation AUC: a learning rate of 2 × 10^−5^, a weight decay of 0.01 and a per-device batch size of 4. We optimized the cross-entropy loss using the AdamW optimizer. Using the full dataset, we fine-tuned the pretrained model using subsamples of the NYU Mortality dataset and evaluated their AUC on the temporal test set. For each size of subsample, we ran five experiments with distinct random seeds (0, 13, 24, 36, 42). For comparison, we looked at the median AUC and the standard deviation of the five experiments.

#### NYUTron + H&P notes for binned comorbidity prediction

We replaced the trained MLM classifier with a randomly initialized linear classifier after the last hidden layer of the pretrained BERT model. We fine-tuned the model end to end using the training set of the NYU Binned Comorbidity dataset for ten epochs, evaluating the validation OVR AUC every half epoch and stopping early with a patience of 5. We used the following hyperparameters from manual tuning based on the validation OVR AUC: a learning rate of 2 × 10^−5^, a weight decay of 0.01 and a per-device batch size of 4. We optimized the cross-entropy loss using the AdamW optimizer. Using the full dataset, we fine-tuned the pretrained model with subsamples of the NYU Binned Comorbidity dataset and evaluated their OVR AUC on the temporal test set. For each size of subsample, we ran five experiments with distinct random seeds (0, 13, 24, 36, 42). For comparison, we looked at the median OVR AUC and the standard deviation of the five experiments.

#### NYUTron + H&P notes for binned LOS prediction

We replaced the trained MLM classifier with a randomly initialized linear classifier after the last hidden layer of the pretrained BERT model. We fine-tuned the model end to end using the training set of the NYU Binned LOS dataset for ten epochs, evaluating the validation AUC every half epoch and stopping early with a patience of 5. We used the following hyperparameters from manual tuning based on the validation OVR AUC: a learning rate of 2 × 10^−5^, a weight decay of 0.01 and a per-device batch size of 4. We optimized the cross-entropy loss using the AdamW optimizer. Using the full dataset, we fine-tuned the pretrained model with subsamples of the NYU Binned LOS dataset and evaluated their AUC on the temporal test set. For each size of subsample, we ran five experiments with distinct random seeds (0, 13, 24, 36, 42). For inference, we combined the last two classes, label 3 (90–99% quantile) and label 4 (>99% quantile) because label 4 was very sparse. For comparison, we looked at the median OVR AUC and the standard deviation of the five experiments.

#### NYUTron + H&P notes for insurance denial prediction

We replaced the trained MLM classifier with a randomly initialized linear classifier after the last hidden layer of the pretrained BERT model. We fine-tuned the model end to end using the training set of the NYU Insurance Denial dataset for ten epochs, evaluating the validation AUC every half epoch and stopping early with a patience of 5. We used the following hyperparameters from manual tuning based on the validation AUC: a learning rate of 2 × 10^−5^, a weight decay of 0.01 and a per-device batch size of 4. We optimized the cross-entropy loss using the AdamW optimizer. Using the full dataset, we fine-tuned the pretrained model using subsamples of the NYU Insurance Denial dataset and evaluated their AUC on the temporal test set. For each size of subsample, we ran five experiments with distinct random seeds (0, 13, 24, 36, 42). For comparison, we looked at the median AUC and the standard deviation of the five experiments.

#### NYUTron + clinical notes for NER

We performed the fine-tuning experiments as follows. For each LLM in Extended Data Table 2, we initialized a HuggingFace token classification model with the LLM as the pretrained checkpoint. We fine-tuned the model using i2b2-2012 NER for ten epochs using the AdamW optimizer with a learning rate of 2 × 10^−5^, a weight decay of 0.01 and a batch size of 4, evaluating every 50 steps and stopping early on the basis of area under the receiver operating characteristic (AUROC) with a patience of 1. This took 20 to 40 min on one node of four NVIDIA 17-GB V100 GPUs. We performed fine-tuning five times with random seeds 0, 13, 24, 36 and 42 and recorded the average and standard deviation of the micro-averaged F1 score (excluding the label for non-entity, ‘O’).

#### NYUTron + MIMIC-III readmission

We performed the fine-tuning experiments as follows: For both NYUTron and BioClinicalBert, we initialized a HuggingFace token classification model with the LLM as the pretrained checkpoint. We fine-tuned the model using MIMIC-III Readmission for ten epoch using the AdamW optimizer with a learning rate of 2 × 10^−5^, a weight decay of 0.01 and a batch size of 16, evaluating every half epoch. We performed fine-tuning five times with random seeds 0, 13, 24, 36 and 42.

### Deployment

The fine-tuned model was converted to a high-performance format (Onnx or TensorRT) and loaded into our deployment platform, an NVIDIA Triton inference engine that interfaces with the NYU Langone EHR through the HLA7 Fast Health Interoperability Resources (FHIR)^37^ interface. For our consideration of performance, security, reliability and interpretability, see Supplementary Information section 5.

Our deployment platform consisted of a modified version of NVIDIA’s Triton Inference Server that we named NYUTriton (pronounced ‘nutrition’ because it is good for the health system). NVIDIA Triton supports GPU-, x86- and ARM CPU-based inferencing and several key features, including dynamic batching, concurrent execution, a highly flexible model specification interface, and the ability to support a wide range of deep learning frameworks and accelerated model formats for maximum throughput. We modified NVIDIA Triton to interface seamlessly with HuggingFace-formatted language models so as to provide a uniform and highly flexible crossover point between our development and production pipelines. Trained models were saved in a standard HuggingFace-style format and converted into Onnx and then TensorRT to obtain sub-millisecond-scale inference results. NYUTriton is hosted on a dedicated inference server that consists of an AMD Threadripper 3960X (24 cores, 3.8 GHz), two RTX 3090 GPUs and 128 GB of DDR5 system memory purchased from Lambda Labs.

Following the signing of discharge summaries in Epic, the HL7 FHIR interface connects with NYUTriton and sends a JavaScript Object Notation (JSON) payload consisting of the discharge summary and metadata specifying the underlying readmission model and sender. NYUTriton preprocesses the text, runs an inference job with the accelerated NYUTron readmission model and returns the model’s inference result to a secondary orchestration server, which writes the result to a database and generates an email to the signing physician.

### Structured baselines

The structured baselines were (1) SAPS2/APACHE2 features + XGBoost for in-hospital mortality prediction, (2) LACE features + XGBoost for readmission prediction, (3) Lisbon Portugal features + XGBoost for binned LOS prediction and (4) claim form features + XGBoost for insurance denial prediction.

For all structured baselines, we used the xgboost library to train an extreme gradient-boosted tree classifier with a binary logistic loss (multiclass softmax loss for more than two classes). We used scikit-learn’s randomized search to search hyperparameters among minimum_child_weight from {1, 5, 10}, gamma from {0.5, 1, 1.5, 2, 5}, subsample from {0.6, 0.8, 1}, col_sample_bytree from {0.6, 0.8, 1.0}, max_depth from {3, 4, 5}, learning_rates from {0.001, 0.01, 0.1, 0.5} and n_estimators from {10, 100, 1000} for 100 iterations based on AUROC score (ovr-auroc score for multiple classes) from threefold cross-validation^38^. We ran each experiment five times with distinct random seeds (0, 13, 24, 36, 42). For mortality, binned comorbidity, binned LOS and insurance denial, we ran the experiment with the full dataset. For readmission, we trained the model using subsamples (*N* ∈ {10^2^, 10^3^, 10^4^, 10^5^, 3.92336 × 10^5^}) of the NYU Readmission–LACE dataset.

### Metrics

We evaluated the five tasks (in-hospital mortality prediction, binned comorbidity index prediction, 30-day all-cause readmission prediction, binned LOS prediction and insurance denial prediction) with AUC for binary classes and OVR AUROC for multiple classes. AUROC is the area under the two-dimensional curve consisting of tuples of the form (TPR, FPR) resulting from different decision thresholds.

We additionally evaluated readmission prediction with the following metrics: TPR, FPR, precision, recall and F1 score, all of which have a range of [0, 1]. We evaluated NER using a micro-averaged NER F1 score. The NER F1 score is similar to the normal F1 score except that the non-entity label ‘O’ is excluded for calculation.

### Baseline algorithms for retrospective study

We compared NYUTron against physicians. We worked with six physicians with different levels of seniority: three attending physicians and three residents. The physicians were asked to review discharge summaries and predict whether the described patient would come back to the hospital within 30 days.

We compared NYUTron against four other LLMs and two machine learning models. ‘random-init’ is a BERT-base uncased model with randomly initialized parameters. ‘web-wiki’ is a BERT-base uncased model that is pretrained using web text (from the BookCorpus dataset^39^) and Wikipedia articles (from the English Wikipedia dataset^40^). ‘web-wiki+bio’ is a BERT model pretrained using web text, Wikipedia articles, PubMed abstracts^41^ and PubMed Central (PMC) full articles^42^. ‘web-wiki+bio+clinical’, or gatortron-og^43^, is a Megatron-BERT^44^ model pretrained using web text, Wikipedia articles, PubMed abstracts, PMC full articles, MIMIC-III notes and de-identified clinical notes from University of Florida Health. ‘lace+xgb’ reads structured LACE features (from a traditional clinical prediction rule) with an extreme gradient-boosted tree model^14^. ‘tf-idf+xgb’ reads corpus-level bag-of-words features with an extreme gradient-boosted tree model. For detailed statistics and examples of the pretraining corpora, see Extended Data Table 2 and Extended Data Fig. 3.

### Comparison with physicians

We randomly sampled 20 discharge notes from the random test set and asked six doctors with different seniority to predict whether the patient would come back within 30 days. The six physicians included three attending neurosurgeons, two neurosurgery residents and one ICU resident.

We used REDCap to perform the survey and gave physicians unlimited time. The survey was structured as follows: for each case, we asked “Will this person be admitted within 30 days?”, followed by the discharge summary. The physician could choose to answer “yes” or “no”. If the patient came back within 30 days, we had three follow-up questions to assess the characteristics of the subsequent readmission. First, we asked “Is this readmission related to the prior discharge?”, followed by the H&P note of the subsequent readmission. The physician could answer “yes”, “no”, “partial” or “does not meet Medicare criteria for 30-day readmission”. The second follow-up question was “Is this readmission preventable?”, to which the physician could answer “yes”, “no” or “partial”. The third follow-up question, “Any comments?”, had a free-text response where the physician could explain why the readmission was partially related to the prior discharge or why the readmission was partially preventable.

To collect NYUTron’s predictions, we used the text classification pipeline from HuggingFace to perform inference on the 20 discharge notes. For each discharge note, the pipeline output a predicted probability for readmission. We converted this predicted probability to a binary label with a threshold of 0.07 (a predicted probability no less than 0.07 was converted to a positive label). We chose 0.07 as the decision boundary because it was the minimum threshold that gave us above 80% validation recall among the thresholds {0.01 × *n* : *n* ∈ {1, ..., 90} (the 80% criterion was chosen on the basis of clinical applicability). See Extended Data Fig. 8c for NYUTron’s calibration curve.

### Comparison with other language models

#### Discharge notes + other LLMs for readmission prediction

The dataset, hyperparameters, and evaluation and software libraries for fine-tuning other LLMs were the same as when fine-tuning NYUTron. The pretrained LLMs were constructed as follows: random-init is a BERT-base uncased model with reset parameters. web-wiki is a BERT-base uncased model. web-wiki+bio is a dmis-lab/biobert-base cased v1.2 model. web-wiki+bio+clinical was Gatortron-og downloaded from NVIDIA NGC and converted to a HuggingFace checkpoint using convert megatron bert checkpoint.

#### Clinical notes + other LLMs for NER

The dataset, hyperparameters, and evaluation and software libraries for fine-tuning other LLMs were the same as for fine-tuning NYUTron. The pretrained LLMs were the same as the baseline LLMs for predicting readmission from discharge notes.

### Comparison with machine learning models

#### LACE features + XGBoost for readmission prediction

Using the NYU Readmission–LACE dataset, we used the xgboost library to train an extreme gradient-boosted tree classifier with binary logistic loss with hyperparameter search. We used scikit-learn’s randomized search to search among minimum_child_weight from {1, 5, 10}, gamma from {0.5, 1, 1.5, 2, 5}, subsample from {0.6, 0.8, 1}, col_sample_bytree from {0.6, 0.8, 1.0}, max_depth from {3, 4, 5}, learning_rates from {0.001, 0.01, 0.1, 0.5} and n_estimators from {10, 100, 1000} for 100 iterations on the basis of AUROC score on the validation set^37^. We trained the model using subsamples (*N* ∈ {10^2^, 10^3^, 10^4^, 10^5^, 3.92336 × 10^5^}) of the NYU Readmission–LACE dataset and evaluated their AUROC on the temporal test set. For each size of subsample, we ran five experiments with distinct random seeds (0, 13, 24, 36, 42). For comparison, we looked at the median AUROC and the standard deviation of the five experiments.

#### XGBoost + TF-IDF for readmission prediction

We transformed the text from the NYU Readmission dataset into tf-idf (term frequency–inverse document frequency) embeddings and used an xgboost classifier with binary logistic loss to predict readmission. We used raytune^45^ to search hyperparameters, including max_tf-idf features from {512, 5000}, max_depth from a quantized random integer of 3 to 16 with an interval of 4, learning_rate from a log uniform distribution from 10^−2^ to 10^−1^, gamma from a quantized uniform distribution from 0 to 12 with an interval of 4, minimum_child_weight from a quantized uniform distribution from 0 to 8 with an interval of 4, reg lambda from a quantized uniform distribution from 0 to 10 with an interval of 2, colsample_bytree from a uniform distribution from 0.7 to 1, scale pos weight from a quantized uniform distribution from 0 to 50 with an interval of 10 and n_estimator from a quantized integer distribution from 50 to 300 with an interval of 50. We trained the model using subsamples (*N* ∈ {10^2^, 10^3^, 10^4^, 10^5^, 3.92336 × 10^5^}) of the NYU Readmission dataset and evaluated their AUROC on the temporal test set. For each size of subsample, we ran five experiments with distinct random seeds (0, 13, 24, 36, 42). For comparison, we looked at the median AUROC and the standard deviation of the five experiments.

### Comparison of multi-site pretraining and fine-tuning

We compared NYUTron with its four variants (pretrained and fine-tuned using data from different sites): (1) NYU Notes–Manhattan + NYU Readmission–Manhattan, (2) NYU Notes–Manhattan + NYU Readmission–Brooklyn, (3) NYU Notes–Brooklyn + NYU Readmission–Brooklyn and (4) NYU Notes–Brooklyn + NYU Readmission–Manhattan. The hyperparameters and evaluation and software libraries for fine-tuning NYUTron variants were the same as for fine-tuning NYUTron.

### Analysis of prospective performance

On the basis of the temporal test performance in the retrospective study, we selected a fine-tuned model with a decision threshold of 0.07 for use in the prospective trial.

#### Comparison of mortality rate and LOS

To assess the condition of the readmitted patients who were correctly predicted (*n* = 3,298), we compared their in-hospital mortality rate and length of hospitalization with that of patients who were admitted in the same period. We collected data on patients who were admitted from February to May 2022 (*n* = 30,548) and compared their in-hospital mortality rate and LOS with that of the readmitted patients caught by NYUTron from January to April 2022. We used two-sided Welch’s *t* tests (with the null hypothesis that the two groups had the same average) to assess the statistical significance of our comparison^46^.

#### Assessing NYUTron’s clinical impacts with physician review

We performed a post hoc analysis of readmitted patients in the prospective cohort to better understand model performance in a real-world environment and in anticipation of creating targeted interventions based on model outputs. One hundred readmitted patients were sampled from the five largest departments at NYU Langone by patient volume: internal medicine, pediatrics, general surgery, obstetrics and gynaecology, and haematology and oncology. Each department contributed 20 cases, with 10 cases having the highest predicted probabilities in that department and 10 cases having the lowest predicted probabilities. All cases had their encounter IDs logged for their index discharge and readmission on a secure online platform. A standardized questionnaire was constructed for manual review asking whether the readmission was planned, whether the readmission met CMS criteria for a penalized 30-day readmission, whether the readmission was preventable, whether an adverse event occurred on readmission, whether any adverse events were preventable and whether the reviewing physicians had any comments on the case. A team of ten physicians from internal medicine and neurosurgery were randomly assigned cases to be reviewed in pairs, with any disagreement between the reviewers adjudicated by a third physician reviewer. To determine whether a readmission was preventable, the reviewer looked at the discharge note of the inference encounter and the H&P note of the readmission encounter.

### Ethical approval

Our research was approved by the NYU Langone institutional review board as ‘s21-01189 NYUtron’, and the methods were carried out in accordance with the institutional review board’s relevant guidelines and regulations.

### Reporting summary

Further information on research design is available in the Nature Portfolio Reporting Summary linked to this article.

## Online content

Any methods, additional references, Nature Portfolio reporting summaries, source data, extended data, supplementary information, acknowledgements, peer review information; details of author contributions and competing interests; and statements of data and code availability are available at 10.1038/s41586-023-06160-y.

### Supplementary information

Supplementary Information .

Reporting Summary

## Figures and Tables

**Extended Data Fig. 7:**
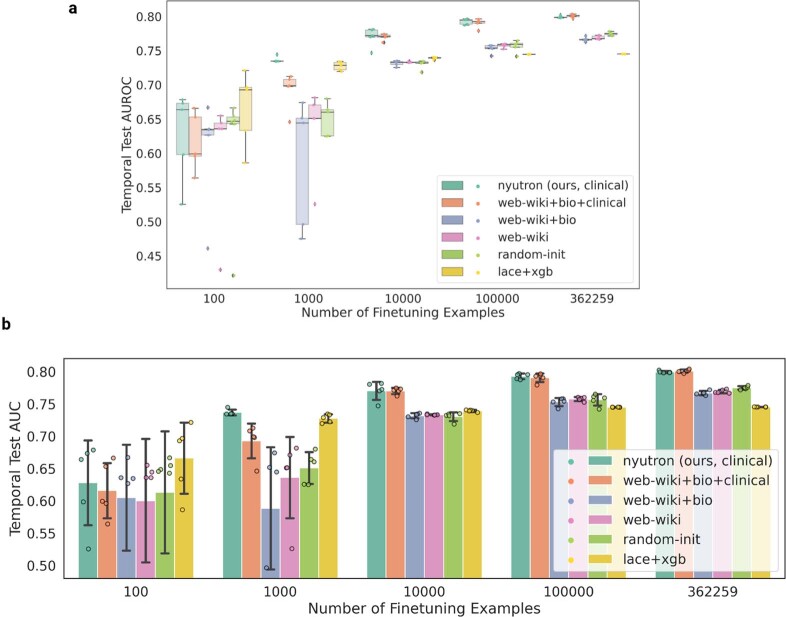
Detailed statistics of the comparison between language models and lace+xgb. **a**, A box plot with individual data points. For each model, 5 experiments were run using random seeds 0, 13, 24, 36, 42. The centerline of the box plot indicates the median. The upper line of the box indicates the first quantile. The lower line of the plot indicates the last quantile. The whisker extends to 1.5 times the interquartile length and the diamonds indicate outliers. **b**, A bar plot that shows the mean and standard deviation. The height of the bar indicates the mean across 5 experiments and the length of the black vertical line indicates the standard deviation.

**Extended Data Table 1:**
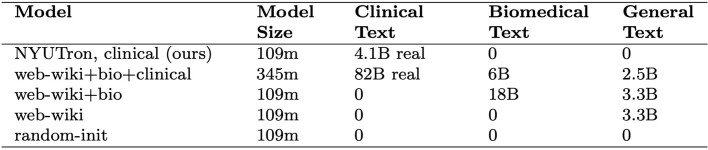
Detailed statistics of datasets

We built a comprehensive pretraining dataset (NYU Notes) with two site-specific variants (NYU Notes - Manhattan/Brooklyn) as discussed in the Methods section. For readmission prediction, we also built a finetuning dataset (NYU Readmission) with two site-specific variants (NYU Readmission Manhattan/Brooklyn), one structured-data variant (NYU Readmission - LACE), and a deployment test set (NYU Readmission - Deployment) that was sampled in real-time as part of our prospective trial. To test the breadth of NYUTron’s applicability, we added 4 tasks (NYU Mortality, NYU Binned LOS, NYU Comorbidity, NYU Insurance denial) with their respective structured-data variant (NYU Mortality - SAPS2+APACHE2, NYU Binned LOS - Lisbon Portugal, NYU Insurance Denial - Claim forms). NYU Comorbidity has no structured-data variant because the task is to impute comorbidity index with the lack of structured icd codes. Finally, we have a Named Entity Recognition (NER) dataset for testing how well NYUTron generalizes to different clinical predictive tasks using non-NYU data.

**Extended Data Table 2:**
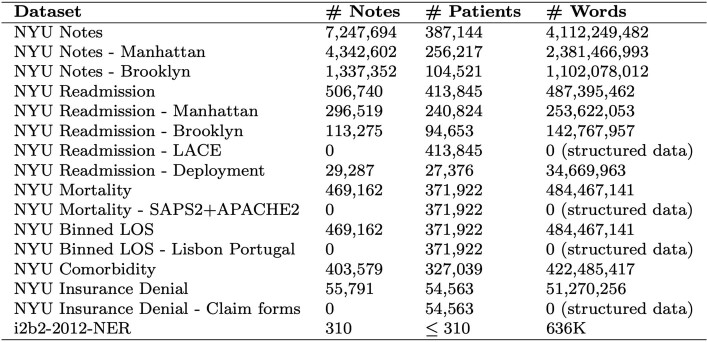
Sizes and pretrain corpora for LLMs

We test 6 types of LLMs with different model sizes and different pretraining corpora. We list out the various corporate here as well as model parameter counts to facilitate ease of comparison. We also note that one key distinction between web-wiki+bio+clinical and NYUTron, clinical is that the former was stripped of identifying information while the latter was not.

## Data Availability

The clinical data used for the pretraining, fine-tuning, validation and test sets were collected from the NYU Langone Health System EHR maintained by the NYULH Datacore team. Text data were stripped of rich-text features and directly included in the dataset ‘as is’ and were augmented with structured features where noted. These data consist of the production medical records of NYU Langone and cannot be made publicly available. Researchers may obtain a limited de-identified dataset (or a test subset) from NYU Langone Health System by reasonable request and subject to local and national ethical approvals. We also used publicly available i2b2-2012 (https://portal.dbmi.hms.harvard.edu/projects/n2c2-nlp/) and MIMIC-III (https://physionet.org/content/mimiciii/1.4/) datasets.

## Code availability

We used sql and Python 3.8.13 to collect data from the NYU Langone EHR. We used REDCap 12.4.31 to collect physician responses. This work used several open-source libraries, including HuggingFace Transformers 4.19.2, Datasets 2.2.2, Evaluate 0.1.1, wandb 0.12.17, matplotlib 3.5.2, seaborn 0.12.2, pandas 1.4.2, ray 2.0.0, sklearn 1.1.1, deepspeed 0.8.0+384f17b, NVIDIA Apex, XGBoost 1.6.1 and nltk 3.6.3. Our experimental framework involved the use of these libraries and, in some cases, modification of them. We will release code to replicate the pretraining, fine-tuning and testing of the models described in this paper at the time of publication (code for experiments available at https://github.com/nyuolab/NYUTron, preprocessing code for i2b2-2012 available at https://github.com/nyuolab/i2b2_2012_preprocessing). We include detailed methods and implementation steps in the Methods and Supplementary Information to allow for independent replication.
