# Supplementary material for: Health system-scale language models are all-purpose prediction engines
Source: Nature. 2023 Jun 7;619(7969):357–62. doi: 10.1038/s41586-023-06160-y (PMC10338337; doi:10.1038/s41586-023-06160-y)
Supplement: Supplementary file 1 — . [file 41586_2023_6160_MOESM1_ESM.pdf]

nature portfolio

https://doi.org/10.1038/s41586-023-06160-y

Supplementary information

Health system-scale language models are all-purpose prediction engines

In the format provided by the
authors and unedited

Supplementary Information: NYUTron

Lavender Yao Jiang[1,2], Xujin Chris Liu[1,3], Nima Pour Nejatian[4], Mustafa Nasir-Moin[1], Duo Wang[5], Anas Abidin[4], Kevin Eaton[6], Howard Riina[1], Ilya Laufer[1], Paawan Punjabi[6], Madeline Miceli[6], Nora C. Kim[1], Cordelia Orillac[1], Zane Schnurman[1], Christopher Livia[1], Hannah Weiss[1], David Kurland[1], Sean Neifert[1], Yosef Dastagirzada[1], Douglas Kondziolka[1], Alexander T.M. Cheung[1], Grace Yang[2], Ming Cao[2], Mona Flores[5], Anthony B. Costa[5], Yindalon Aphinyanaphongs[5,8], Kyunghyun Cho[2,7,10,11] and Eric Karl Oermann[1,2,9*]

[1]Department of Neurosurgery, NYU Langone Health, 450 First Avenue, New York City, 10019, NY, USA.

[2]Center for Data Science, New York University, 60 5th Ave, New York, 10011, NY, USA.

[3]Electrical and Computer Engineering, Tandon School of Engineering, 6 MetroTech Center, New York City, 11201, NY, USA.

[4]NVIDIA, 2788 San Tomas Expressway, Santa Clara, 95051, CA, USA.

[5]Predictive Analytics Unit, NYU Langone Health, 450 First Avenue, New York City, 10019, NY, USA.

[6]Department of Internal Medicine, NYU Langone Health, 450 First Avenue, New York City, 10019, NY, USA.

[7]Prescient Design, Genentech, 149 5th Ave. 3rd floor, New York, 10010, NY, USA.

[8]Department of Population Health, NYU Langone Health, 450 First Avenue, New York City, 10019, NY, USA.

[9]Department of Radiology, NYU Langone Health, 450 First Avenue, New York City, 10019, NY, USA.

[10]Courant Institute of Mathematical Sciences , New York University, 251 Mercer Street, New York City, 10012, NY, USA.

[11]Canadian Institute for Advanced Research , 661 University Ave, Toronto, M5G1M1, ON, Canada.

Contents

1 Previous Works **3**

2 Details on prediction tasks **4**
2.1 Readmission Prediction 4
2.2 Insurance Denial Prediction 7
2.3 Comorbidity Imputation 7

3 Comparison of NYUTron v.s. other models **8**
3.1 Comparison of Implementation Complexity: NYUTron v.s. FHIR+RNN 8
3.2 Comparing the multifacted complexity of NYUTron v.s. traditional clinical predictive model 9

4 Robustness and generalization **10**
4.1 Clinical language model is able to generalize across different health systems through local finetuning 10
4.2 Text data is not necessarily less robust than structured data . . 10
4.3 Potential explanations of the subgroup discrepancies 11

5 Deployment platform -NYUTriton **12**

6 References **14**

1 Previous Works

Traditional clinical prediction rules that have existed for decades relies on a small set of hand-selected structured features. Three well-known examples are CHADS2 score for atrial fibrillation stroke risk, Child-Pugh score for cirrhosis mortality, and Well's criteria for pulmonary embolism [1–4]. An example for readmission prediction is the LACE score, which uses 4 features: Length of stay, Acuity of readmission, Comorbidity index and the number of recent visits to the Emergency department.

Approaches that are based on **traditional machine learning models** learns from a set of automatically selected structured features [5, 6]. For example, Duke University Health System use regression with L1 regularization to select features from patient age, diagnosis variables, laboratory variables, medications, order types and utilization variables [7]. Their readmission prediction model is a regression model on the selected features. (See Supplemental 3.2 for a complexity comparison with NYUTron.)

Another approach represents clinical notes with embeddings from **traditional NLP models**. For example, to predict readmission from discharge notes, [8, 9] passes the LDA (Latent Dirichlet allocation) / TF-IDF (Term frequency - inverse document frequency) embeddings of discharge notes to an 2-class SVM (support vector machine).

With the advent of EHR, anothger approach for clinical prediction is to apply deep learning to high-dimensional structured EHR data. We will refer to them as **"structured EHR"** approach. For example, [10] takes in the entire EHR associated to a patient using the FHIR format (with task-specific labels) and train an RNN with end-to-end.

Recently, researchers start to use clinical texts from electronic health record to train **clinical language models**. Examples of encoder-based models include ClinicalBERT [11] (which further pretrained BERT using ICU notes from MIMIC-III, and subsequently finetuned to predict readmission from ICU notes) and Gatortron [12] (which pretrained a 345-million parameter Megatron-BERT model using notes from the University of Florida Health and finetuned for 5 clinical NLP tasks including named entity recogntion). Examples of large autoregressive models include PubmedGPT [13], a 2.7 billion parameter model that performs well on biomedical question answering.

The gap: Traditional clinical prediction rules and traditional machine learning models rely on structured data, which is often missing from hospital EHRs. Traditional NLP models do not benefit from pretraining with an increasing amount of unlabelled clinical notes. Structured EHR approaches also faces issues with missing structured features, not leveraging the vast amount of unlabelled data, and the high cost of implementation. (See Supplemental 3.1 for an example.) While recent studies on clinical language models show potential for translating advances in NLP to improving quality of healthcare, they are limited in that (1) they evaluate on a small subset of patient population (e.g., ICU patients from MIMIC-III; patients with strokes)

and (2) they did not perform prospective evaluation, which better resembles the deployment setup by hardening the model and testing it outside the development environment.

Our contribution: We are the first to pretrain a large language model on an entire health system's identified clinical notes and deploy the finetuned model for a prospective trial for all patients. We show that our clinical language model has a wide breadth of applicability to several clinical and operational tasks, as demonstrated by their improved performance over traditional structured data baselines. On a specific clinical predictive task (readmission prediction), we showed the benefit of pretraining with clinical texts, the cross-site generalizability through local finetuning, and the deployability with a prospective, non-interventional, single-arm trial.

2 Details on prediction tasks

2.1 Readmission Prediction

Readmission prediction is a **classic, well-studied clinical predictive problem with practical clinical significance**. Readmission puts patients at risk medically and financially, and reducing readmission rates could improve the quality of care. Every year, 1.15 billion patients are discharged globally, and in the United States 14% of discharged patients are ultimately readmitted. Nationally, readmitted patients are, on average, associated with an extra cost to providers of $15,000 [14]. To reduce preventable readmissions, the Center for Medicare and Medicaid Services (CMS) launched a hospital readmission reduction program that decreases payments to hospitals according to the rate of unplanned 30-day readmission. Due to the significance of this problem both clinically and operationally, several attempts [7, 11] have been made to build and deploy 30-day readmission models by both health systems and EHR vendors with varying results.

We estimated **the scale of readmission prediction problem** as follows: 1) To estimate the number of patients discharged annually, we use the number of hospital discharges per one thousand person in OECD countries in 2017. OECD countries have 17.9% of the world's 7.52 billion populations, and 154 hospital discharges per 1000 population [15, 16]. Assuming that the discharge rate is similar in non-OECD countries, we estimate the total number of hospital discharge in 2017 around the world as $7.52 \cdot \frac{154}{1000} \approx 1.16$ billion discharges. 2) To investigate how often discharged patients get readmitted, we use the readmission rate and cost from United States. In 2018, United States has a 14% readmission rate with an average readmission cost of $15,200 [14].

To reduce preventable readmission in United States, Center for Medicare and Medicaid Services (CMS) launched a **Hospital Readmission Reduction Program** (HRRP). Starting from October 1, 2012, the U.S. government reduces a maximum of 3% of payments to hospitals with excessive readmission, as measured by "30-day risk-standardized unplanned readmission" [17].

Discharge Notes contain signals for readmission prediction. A word cloud of discharge notes in NYU Readmission in shown in Figure 1. We constructed word clouds based on non-readmitted and readmitted labels from NYUTron where a word with a larger log odds ratio has a larger font size. On the left, non-readmitted patients seem to have milder diseases such as "pancreatitis" and have "friends" who can pick them up upon discharge. On the right, readmitted patients have more serious disease such as "lymphoma", which requires frequent hospital visits for chemotherapy and radiotherapy.

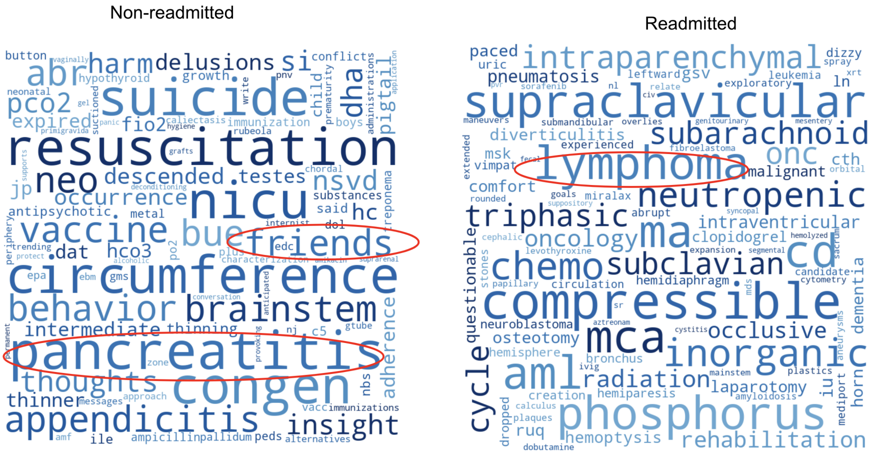

Fig. 1: The word cloud of discharge notes from non-readmitted patients (left) and readmitted patients (right)

In this paper, we **define readmission as 30-day all-cause readmission**. That is, we say a patient is readmitted if there is a subsequent admission within 30 days.

Our definition of "readmission" does not solely consist of preventable readmission (the ones that people care most about, or the yellow box after L3, as shown in Figure 2). For example, if a patient had a successful brain surgery, went home and fell over the stairs, we will still say the patient has an "all-cause 30-day readmission" although it is not preventable.

In an ideal scenario, we should finetune with the "unplanned, preventable 30-day readmission" label. However, we do not have this label in our database, so we decided to use a looser label (from L1 in Figure 2) and leave the rest of the decisions (L2 and L3) for the physicians. Our L1 label covers the set of all "unplanned, preventable 30-day readmission": that is, if a case has a positive L3 label, it must have a positive L1 label.

To get more precise labels, we need to recruit a team of experienced physicians to manually annotate each one of the 506,740 cases, with potential disagreement over which cases are preventable. This annotation is expensive with ambiguity over the "preventable" label, and we think the costs outweigh

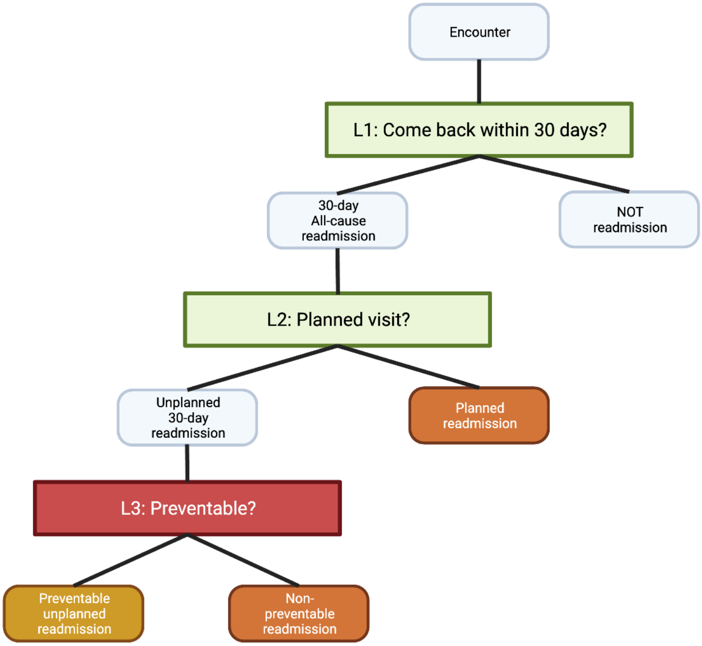

Fig. 2: Three levels for predicting readmission. We choose to use the L1 label because obtaining L2 and L3 labels is expensive. At deployment, the physicians can use their judgment for L2 and L3 to filter out the false positives for "preventable, unplanned readmission

the benefits. To elaborate, our current readmission prediction model (fine-tuned with L1 label) will alert unplanned, preventable 30-day readmission with some false positives (orange boxes: nonpreventable cases and planned cases). At deployment, the physician can use their judgement to filter out the false positive. For example, if the physician got alerted for a case with 3-day followup, we assume the physician will ignore the alert because they know the predicted readmission is planned. If we train a model with L3 label, the benefit is that there will be fewer false positive, and the costs is expensive annotation and potentially missing preventable cases from the ambiguity of annotating "preventable" cases.

Practical significance of performance improvement: Given a large patient cohort, every 0.01% improvements could positively affect the health of real patients. For example, suppose we improve the recall of readmission from 78% to 80% for a cohort of 27,376 patients from January to April of 2022 (the size of NYU Readmission - Deployment, shown in Extended Data Table 1) with readmission rate of 10%. That means an extra 55 high-risk

patients would be identified prior to discharge. Suppose 27% of the patients' readmission are preventable (from Main Text Figure 4b), then we could stop around 15 patients from coming back to NYU Langone with interventions (e.g., scheduling followup calls, delaying discharge, at-home visits). Even small improvement could prevent real patients from suffering from readmission, on which they are six times more liketly to die and stay three days longer, with an additional cost of $15,000 per patient.

2.2 Insurance Denial Prediction

For patients with insurance, hospitals receive compensation from their insurance companies by submitting insurance claims that document the details of the visit such as procedures done and neccessity of the procedure. However, the claimed amount does not always get fully reimbursed, which increases the operating costs of a health system.

The task of insurance denial is to predict (at the point of care) whether the claim associated with that visit will be denied by the insurance providers. This would help reduce unnecessary out-of-pocket costs of the patients and the financial stress of the health system.

In our dataset, we consider three possible outcomes of a insurance claim: (1) the claim is directly approved, (2) the claim is initially rejected, but approved upon appeal, (3) the claim is initially rejected, and still rejected upon appeal.

We say a claim is "*initially denied*" for outcomes (2) and (3), and a claim is "directly approved" for outcome (1). We say a claim is "*eventually denied*" for outcomes (3), and a claim is "eventually approved" for outcome (1) and (2).

We perform four types of prediction using the same method described in Method "NYUTron + H&P Notes for Insurance Denial Prediction":

1. Using NYUTron Insurance Denial dataset, predict whether a claim is initially denied from H&P notes (shown in Main Text Figure 2c, AUC 87.2%±0.246%).
2. Using NYUTron Insurance Denial - D/C Notes dataset, predict whether a claim is initially denied (AUC 87.71%±0.188%).
3. Using NYUTron Insurance Eventual Denial - H&P Notes dataset, predict whether a claim is eventually denied(87.54%±0.312% AUC).
4. Using NYUTron Insurance Eventual Denial - D/C Notes dataset, predict whether a claim is eventually denied(AUC 88.0%±0.313%).

2.3 Comorbidity Imputation

Charlson comorbidity index (CCI) quantifies the severity of a paitent's health condition based on the patient's history of chronic disease and severe condition. The index chooses a set of chronic diseases (e.g., congestic heart failure, liver disease) and assigns a positive score for each chronic disease. The final index sums over all the score, and a larger index indicates a more severe health condition. The index can help physicians predict patient outcomes.

The conventional calculation of CCI requires data collection and manual entry. Using EHR, we can automate the process by first identifying the history of chronic disease using ICD (International Classification of Diseases) diagnosis codes, and then assigining scores based on the ICD codes.

However, the ICD codes are missing for certain patients (in our case, 22% of the encounter). For example, patients who transferred from an external health system with a separate EHR will have no past ICD codes. In this case, we want to impute the comorbidity index. This setting is different than common imputation tasks, in that *not partial, but all* structured data are missing. Motivated by the richness of care-relevant information in clinical notes, we propose to impute CCI using clinical notes and language models.

3 Comparison of NYUTron v.s. other models

3.1 Comparison of Implementation Complexity: NYUTron v.s. FHIR+RNN

To illustrate NYUTron's benefit of low-cost implementation and low-resistance deployment, here we show a comparison of developing and deploying (1) FHIR+RNN model used in [10], as outlined in their supplementary materials, v.s. (2) NYUTron.

Here are the 7 steps that would be required for preparing data to the FHIR format:

1. Joining data: We need to include at least the following 19 tables with 1207 total columns, as shown in Table 1. We need to write multiple sql scripts to join them together.
2. Data cleaning: we need to manually examine and remove fields of data with mostly null values, and fields that contain care-irrelevant information (e.g., billing). Some examples include 'isdeleted' and 'lastupdatedinstant'.
3. Value mapping: we need to map text fields for diagnosis into standardized ICD-9 or ICD-10 codes.
4. Processing flowsheets: we need to sort vital signs and nursing documentation by the entry time.
5. Convert to FHIR format: for each patient, create a json file that captures their entire medical history as a sequence of events, represented by various features.
6. Further processing based on feature types. For example, if the feature is numeric value, we need to either concatenate the value with their units, or convert this value to its quantile representation. For the delta time between events, we need to choose between rounding, capping, log scale, and discretization with buckets.
7. Choosing the embedding size for each feature: either choose it as the number of unique values for that features, or do a hyperparameter search. (For high-dimensional features, doing a hyperparameter search for each feature is very expensive)

Name	# columns
encounters	123
patients	133
medication	25
medication_order	135
medication_event	59
lab_test	164
lab_component_result	57
diagnosis	33
diagnosis_event	48
diagnosis_terminology	22

Name	# columns
procedure	50
procedure_event	56
procedure_order	65
procedure_terminology	15
surgical_procedure_event	48
dental_procedure_event	52
provider	70
clinical_note	39
clinical_note_text	13

Table 1: Minimal Tables in NYU Datalake Required for the FHIR+RNN Approach. Table names were pseudonymized for license compliance.

As a comparison, our **language model** based approach has a low-resistance data preparation with minimal manual processing and requires just 2 step:

1. Joining data: collect clinical notes from encounters, clinical_note, and clinical_note_text. The queried data has 2 columns: encounterkey and text. For self-supervised pretraining, the data preparation is finished. For supervised finetuning, we can additionally add a column of labels.
2. Preprocessing text: train a tokenizer from the pretraining text and tokenize the finetuning text.

Apart from the difficulty of the data preparation, the approaches based on high dimensional structured data have the additional problem of being challenging to deploy. Integration with FHIR data requires the full interoperability of a potential EHR system with FHIR. While the Office of the National Coordinator for Health Information Technology has mandated FHIR interoperability by end-of-year 2022, challenges remain in real world support and compatibility. With LLM based approaches, integration can still be achieved using FHIR, but can be as simple as copying and pasting as the only required input is free text.

A future research question is whether there exists a trade-off between language model's better deployability and potential losses in performance compared to models that relies on more complicated data structure. Such question remains difficult to investigate due to existing data infrastructure and software ecosystem in hospitals, as evidenced in the very limited use of advanced ML model in real clinical environments [18–20].

3.2 Comparing the multifacted complexity of NYUTron v.s. traditional clinical predictive model

NYUTron is more *computationally-complex* and *storage-complex* than traditional clinical predictive model because it performs more computations and has more stored parameters.

NYUTron is less *data-complex* than traditional clinical predictive model because it requires less data fusing, imputation, and feature engineering. We demonstrated this in our rapid prototyping and implementation of four additional tasks under 1 week.

NYUTron is less *deployment-complex* than traditional clinical predictive model, because they enable real-time inference as physicians write notes and require fewer labelled examples. With clinical LLMs, physicians can get real-time predictions as soon as they sign their notes in the EHR.

4 Robustness and generalization

4.1 Clinical language model is able to generalize across different health systems through local finetuning

Here we give two examples of across-health-system generaliation through local finetuning.

The first example is Gatortron-og (from University of Florida Health) generalizes to NYU Readmission (from NYU Langone Health). In Main Text Figure 3b, the orange line comes from finetuning Gatortron-og, a language model pre-trained with a mix of clinical text (notes from University of Florida Health) and non-clinical text (web text, wikipedia, pubmed abstracts). The finetuning data is NYU Readmission, which contains discharge notes from NYU Langone Health. The figure shows that with 100 and 1000 examples, Gatortron has a lower AUC than NYUTron. However, Gatortron catches up to NYUTron after 10,000 local finetuning examples.

The second example is NYUTron (from NYU Langone Health) generalizes to MIMIC-III Readmission (from Beth Israel Deaconess Medical Center in Boston). We finetuned and tested NYUTron on the MIMIC-III readmission dataset, which consists of de-identified discharge notes from the Beth-Israel's ICU with binary labels for 30-day all-cause readmission. We compared NYUTron with BioClinicalBERT[21], whose pretraining data covers the MIMIC notes. Extended Data Figure 4 shows that at 1000 samples, NYUTron has a 3.58% higher median AUC than BioClinicalBERT (57.22% v.s. 53.64%). At 10,000 samples, NYUTron has a 6.42% higher median AUC than BioClinicalBERT (65.56% v.s. 59.14%). Using the full dataset (42,180 samples), NYUTron has a 3.8% higher median AUC than BioClinicalBERT (67.04% v.s. 63.24%).

4.2 Text data is not necessarily less robust than structured data

Main Text Figure 3(c)(d) shows that NYUTron is "non-robust" to changes in deployment site in the sense that: when the model is pretrained on one site, but finetuned and tested on the other site, there is a performance drop compared to doing everything locally.

However, we hypothesize that text-based model are not necessarily *less* robust than structured-data-based model. To show this, we ran the same "Manhattan-versus-Brooklyn" experiments using site-specific variants of NYU Readmission - LACE. The result is shown in Table 2. For brevity, here we focus on the results of Manhattan test and discuss 3 findings.

Trained on / Tested on	Brooklyn	Manhattan
All	57.18% ± 0.319%	62.70% ± 0.345%
Brooklyn	58.37% ± 1.19%	63.11% ± 1.61%
Manhattan	58.11% ± 0.0213%	64.62% ± 0.0824%

Table 2: Manhattan v.s. Brooklyn readmission prediction experiment using lace+xgb

First, when the structured data based model is trained in Brooklyn, and tested in Manhattan (63.11% median AUC), there is also a performance drop (1.51% AUC, or 2.34% relative percentage drop) compared to doing everything locally (model trained and tested in Manhattan has 64.62% AUC).

Second, the performance drop from structured data model is not necessarily smaller than the performance drop from text data model. For example, Main Text Figure 3c shows that when NYUTron is pretrained in Brooklyn, but finetuned and tested in Manhattan (84.11% AUC), there is a performance drop of 0.68% AUC compared to doing everything locally (84.79% AUC), or 0.80% relative percentage drop. Both NYUTron's absolute change (0.68% v.s. 1.51%) and relative change (0.80% v.s. 2.34%) is smaller than the observed drop from lace+xgb. To give another example: Main Text Figure 3c shows that when NYUTron is pretrained in Manhanttan, finetuned in Brooklyn, and tested in Manhattan (80.70% AUC), there is a performance drop of 1.03% AUC compared to doing everything locally (81.73% AUC), or 1.26% relative percentage drop. Both NYUTron's absolute change (1.03% v.s. 1.51%) and relative change (1.26% v.s. 2.34%) is smaller than the observed drop from lace+xgb.

Third, we see that the language models achieve a higher overall AUC (Main Text Figure 3b,c) than lace+xgb (Table 2).

Together, the three finding suggests that NYUTron is not less robust than lace+xgb on readmission prediction, and that it has a better AUC than lace+xgb.

4.3 Potential explanations of the subgroup discrepancies

The complex data generating process of clinical notes (which depends on a variety factors such as social and medical history of patients and providers, interactions between patients and providers, and the norms of our society) makes identifying the causes of subgroup discrepancies shown in Extended Data Figure 5,6 extremely difficult. Here we provide a few speculations and encourage future works in this area.

1. Toxicity and bias in clinical texts. For example, [22] shows that different ethnic groups have different levels of recorded pain. It is possible that the provider's writings were affected by their bias towards different ethnic groups.
2. Inherent difference between subgroup distribution. For example, [23] shows that even using self-reported numerical level of menstrual pain, Australian women have a higher level of pain than Chinese women. It is possible that these two groups naturally have different pain threshold. Another example is that hospitals with higher readmission rates have patients with "more chronic conditions, less education, fewer assets" [24], suggesting that the patient demographics may affect the distribution of readmission.
3. Complex social factors such as systematic racism. For example, it is possible that NYUTron performs worse on predicting black patients' readmission because they have a more complex medical history due to systematic racism, rendering them the more "difficult" cases for prediction.

5 Deployment platform -NYUTriton

Deploying machine learning models in a live healthcare environment carries multiple considerations both technically, clinically, and ethically the full extent of which are beyond the scope of this article. There are numerous essays and editorials on these topics, and we include in our references several which we find particularly lucid on the subject [25–28]. We specifically focus here on our actual experience in deployment of a large language model, NYUTron, in a real-world environment and the unique considerations when working with these large models in terms of performance, security, reliability, interpretability.

Performance is always a major focus of every software engineering project, and here we were significantly aided by the optimizations built into TensorRT and Onnx and nVidia Triton. TensorRT is an accelerated format for deep neural networks that builds in several optimizations to make models faster and more portable. NVIDIA Triton accepts TensorRT or Onnx formatted models, and facilitates their access via its REST API. We chose to run a modified, Dockerized version of NVIDIA Triton in order to take advantage of these optimizations for rapid model inferencing while utilizing on-premises hardware.

Security and monitoring are major concerns in healthcare environments that handle the personal health information of thousands of millions of vulnerable patients. While the present system is naturally suitable to a cloud deployment, and could be done using secured communications to minimize the possibility of data breach, for security purposes we opted to utilize our own internal hardware for model serving. NYUTriton was built to run using docker-compose or as a Helm chart for immediate and scalable deployment via Kubernetes. To facilitate monitoring, NYUTriton was integrated with Prometheus and Grafana to provide continuous monitoring by our engineering team.

Interpretable outputs is one final, additional, consideration when working with LLMs in deployment. While a consideration for medical machine learning algorithms in general, where it has been widely discussed [19, 29], we feel like this bears particular significance in the case of LLMs for two reasons: (1) We propose LLMs as being a potential universal interface for EHR analytics, and with universal inputs comes the added potential of unexpected behaviors, (2) LLMs are particularly complex and black-box in nature. While it is possible to perform sensitivity analysis and to look at attention weighting on inputs to attempt to understand what drives model predictions, we argue that in a real-world medical case interpretability is frequently overrated while evidenced based evaluation is underrated. In our opinion, if LLMs are properly validated in prospective, randomized controlled trials (as are many medical devices), than understanding the inner workings of them is much less relevant. In line with this thinking, we have begun a randomized controlled trial of NYUTron, tied to an intervention, in order to directly assess its performance at delivering a positive impact on patient care.

6 References

[1] Gage, B.F., van Walraven, C., Pearce, L., Hart, R.G., Koudstaal, P.J., Boode, B.S.P., Petersen, P.: Selecting Patients With Atrial Fibrillation for Anticoagulation: Stroke Risk Stratification in Patients Taking Aspirin. Circulation **110**(16), 2287–2292 (2004). https://doi.org/10.1161/01.CIR.0000145172.55640.93

[2] Child, C.G., Turcotte, J.G.: Surgery and portal hypertension. Major Problems in Clinical Surgery **1**, 1–85 (1964)

[3] Pugh, R.N.H., Murray-Lyon, I.M., Dawson, J.L., Pietroni, M.C., Williams, R.: Transection of the oesophagus for bleeding oesophageal varices. British Journal of Surgery **60**(8), 646–649 (2005). https://doi.org/10.1002/bjs.1800600817

[4] Wells, P., Hirsh, J., Anderson, D., Lensing, A.A., Foster, G., Kearon, C., Weitz, J., D'Ovidio, R., Cogo, A., Prandoni, P., Girolami, A., Ginsberg, J.: Accuracy of clinical assessment of deep-vein thrombosis. The Lancet **345**(8961), 1326–1330 (1995). https://doi.org/10.1016/S0140-6736(95)92535-X

[5] Lin, Y.-W., Zhou, Y., Faghri, F., Shaw, M.J., Campbell, R.H.: Analysis and prediction of unplanned intensive care unit readmission using recurrent neural networks with long short-term memory. PLOS ONE **14**(7), 0218942 (2019). https://doi.org/10.1371/journal.pone.0218942

[6] van Walraven, C., Wong, J., Forster, A.J.: LACE+ index: Extension of a validated index to predict early death or urgent readmission after hospital discharge using administrative data. Open Medicine **6**(3), 80–90 (2012)

[7] Gallagher, D., Zhao, C., Brucker, A., Massengill, J., Kramer, P., Poon, E.G., Goldstein, B.A.: Implementation and Continuous Monitoring of an Electronic Health Record Embedded Readmissions Clinical Decision Support Tool. Journal of Personalized Medicine **10**(3), 103 (2020). https://doi.org/10.3390/jpm10030103

[8] Boag, W., Kovaleva, O., McCoy, T.H., Rumshisky, A., Szolovits, P., Perlis, R.H.: Hard for humans, hard for machines: Predicting readmission after psychiatric hospitalization using narrative notes. Translational Psychiatry **11**(1), 32 (2021). https://doi.org/10.1038/s41398-020-01104-w

[9] Orangi-Fard, N., Akhbardeh, A., Sagreiya, H.: Predictive Model for ICU Readmission Based on Discharge Summaries Using Machine Learning and Natural Language Processing. Informatics **9**(1), 10 (2022). https://doi.

org/10.3390/informatics9010010

[10] Rajkomar, A., Oren, E., Chen, K., Dai, A.M., Hajaj, N., Hardt, M., Liu, P.J., Liu, X., Marcus, J., Sun, M., Sundberg, P., Yee, H., Zhang, K., Zhang, Y., Flores, G., Duggan, G.E., Irvine, J., Le, Q., Litsch, K., Mossin, A., Tansuwan, J., Wang, D., Wexler, J., Wilson, J., Ludwig, D., Volchenboum, S.L., Chou, K., Pearson, M., Madabushi, S., Shah, N.H., Butte, A.J., Howell, M.D., Cui, C., Corrado, G.S., Dean, J.: Scalable and accurate deep learning with electronic health records. npj Digital Medicine **1**(1), 18 (2018). https://doi.org/10.1038/s41746-018-0029-1

[11] Huang, K., Altosaar, J., Ranganath, R.: ClinicalBERT: Modeling Clinical Notes and Predicting Hospital Readmission. arXiv (2020)

[12] Yang, X., PourNejatian, N., Shin, H.C., Smith, K.E., Parisien, C., Compas, C., Martin, C., Flores, M.G., Zhang, Y., Magoc, T., Harle, C.A., Lipori, G., Mitchell, D.A., Hogan, W.R., Shenkman, E.A., Bian, J., Wu, Y.: GatorTron: A Large Clinical Language Model to Unlock Patient Information from Unstructured Electronic Health Records. arXiv (2022)

[13] Bolton, E., Hall, D., Yasunaga, Y., Lee, T., Manning, C., Liang, P.: PubMedGPT 2.7BElliot Bolton and David Hall and Michihiro Yasunaga and Tony Lee and Chris Manning and Percy Liang. Technical report, Stanford University (December 2022)

[14] Weiss, A., Jiang, H.: Overview of Clinical Conditions With Frequent and Costly Hospital Readmissions by Payer, 2018. Agency for Healthcare Research and Quality, Rockville, MD (2021). https://pubmed.ncbi.nlm.nih.gov/34460186/

[15] The World Bank: Population, total. https://data.worldbank.org/indicator/SP.POP.TOTL

[16] OECD: Health at a Glance 2019: OECD Indicators (2019). https://doi.org/10.1787/4dd50c09-en

[17] McIlvennan, C.K., J., E.Z., A., A.L.: Hospital readmissions reduction program. Circulation **131**(20), 1796–1803 (2015). https://doi.org/10.1161/CIRCULATIONAHA.114.010270

[18] AIX-COVNET, Roberts, M., Driggs, D., Thorpe, M., Gilbey, J., Yeung, M., Ursprung, S., Aviles-Rivero, A.I., Etmann, C., McCague, C., Beer, L., Weir-McCall, J.R., Teng, Z., Gkrania-Klotsas, E., Rudd, J.H.F., Sala, E., Schönlieb, C.-B.: Common pitfalls and recommendations for using machine learning to detect and prognosticate for COVID-19 using chest radiographs and CT scans. Nature Machine Intelligence **3**(3), 199–217 (2021). https://doi.org/10.1038/s42256-021-00307-0

[19] Kelly, C.J., Karthikesalingam, A., Suleyman, M., Corrado, G., King, D.: Key challenges for delivering clinical impact with artificial intelligence. BMC Medicine **17**(1), 195 (2019). https://doi.org/10.1186/s12916-019-1426-2

[20] Gaube, S., Suresh, H., Raue, M., Merritt, A., Berkowitz, S.J., Lermer, E., Coughlin, J.F., Guttag, J.V., Colak, E., Ghassemi, M.: Do as AI say: Susceptibility in deployment of clinical decision-aids. npj Digital Medicine **4**(1), 31 (2021). https://doi.org/10.1038/s41746-021-00385-9

[21] Alsentzer, E., Murphy, J.R., Boag, W., Weng, W.-H., Jin, D., Naumann, T., McDermott, M.B.A.: Publicly Available Clinical BERT Embeddings. arXiv (2019)

[22] Campbell, C.M., Edwards, R.R.: Ethnic differences in pain and pain management. Pain Management **2**(3), 219–230 (2012). https://doi.org/10.2217/pmt.12.7

[23] Zhu, X., Wong, F., Bensoussan, A., Lo, S.K., Zhou, C., Yu, J.: Are there any cross-ethnic differences in menstrual profiles? A pilot comparative study on Australian and Chinese women with primary dysmenorrhea: Ethnic differences in menstrual profiles. Journal of Obstetrics and Gynaecology Research **36**(5), 1093–1101 (2010). https://doi.org/10.1111/j.1447-0756.2010.01250.x

[24] Barnett, M.L., Hsu, J., McWilliams, J.M.: Patient Characteristics and Differences in Hospital Readmission Rates. JAMA Internal Medicine **175**(11), 1803 (2015). https://doi.org/10.1001/jamainternmed.2015.4660

[25] Chen, P.-H.C., Liu, Y., Peng, L.: How to develop machine learning models for healthcare. Nature Materials **18**(5), 410–414 (2019). https://doi.org/10.1038/s41563-019-0345-0

[26] Matheny, M.E., Whicher, D., Thadaney Israni, S.: Artificial Intelligence in Health Care: A Report From the National Academy of Medicine. JAMA **323**(6), 509 (2020). https://doi.org/10.1001/jama.2019.21579

[27] Yu, K.-H., Kohane, I.S.: Framing the challenges of artificial intelligence in medicine. BMJ Quality & Safety **28**(3), 238–241 (2019). https://doi.org/10.1136/bmjqs-2018-008551

[28] Rajkomar, A., Dean, J., Kohane, I.: Machine Learning in Medicine. New England Journal of Medicine **380**(14), 1347–1358 (2019). https://doi.org/10.1056/NEJMra1814259

[29] Xiao, C., Choi, E., Sun, J.: Opportunities and challenges in developing deep learning models using electronic health records data: A systematic

review. Journal of the American Medical Informatics Association **25**(10), 1419–1428 (2018). https://doi.org/10.1093/jamia/ocy068
