# Supplementary material for: Health system-scale language models are all-purpose prediction engines
Source: Nature. 2023 Jun 7;619(7969):357–62. doi: 10.1038/s41586-023-06160-y (PMC10338337; doi:10.1038/s41586-023-06160-y)
Supplement: Supplementary file 2 — Reporting Summary [file 41586_2023_6160_MOESM2_ESM.pdf]

nature portfolio

Corresponding author(s): Eric Oermann

Last updated by author(s): Mar 28, 2023

Reporting Summary

Nature Portfolio wishes to improve the reproducibility of the work that we publish. This form provides structure for consistency and transparency in reporting. For further information on Nature Portfolio policies, see our Editorial Policies and the Editorial Policy Checklist.

Statistics

For all statistical analyses, confirm that the following items are present in the figure legend, table legend, main text, or Methods section.

n/a	Confirmed	
☐	☒	The exact sample size (n) for each experimental group/condition, given as a discrete number and unit of measurement
☐	☒	A statement on whether measurements were taken from distinct samples or whether the same sample was measured repeatedly
☐	☒	The statistical test(s) used AND whether they are one- or two-sided<br>*Only common tests should be described solely by name; describe more complex techniques in the Methods section.*
☐	☒	A description of all covariates tested
☒	☐	A description of any assumptions or corrections, such as tests of normality and adjustment for multiple comparisons
☐	☒	A full description of the statistical parameters including central tendency (e.g. means) or other basic estimates (e.g. regression coefficient) AND variation (e.g. standard deviation) or associated estimates of uncertainty (e.g. confidence intervals)
☐	☒	For null hypothesis testing, the test statistic (e.g. F, t, r) with confidence intervals, effect sizes, degrees of freedom and P value noted<br>*Give P values as exact values whenever suitable.*
☒	☐	For Bayesian analysis, information on the choice of priors and Markov chain Monte Carlo settings
☒	☐	For hierarchical and complex designs, identification of the appropriate level for tests and full reporting of outcomes
☒	☐	Estimates of effect sizes (e.g. Cohen's d, Pearson's r), indicating how they were calculated

Our web collection on statistics for biologists contains articles on many of the points above.

Software and code

Policy information about availability of computer code

Data collection	We used sql and Python 3.8.13 to collect data from NYU Langone EHR. We downloaded MIMIC-III dataset (https://physionet.org/content/mimiciii/1.4/) and i2b2-2012 dataset (https://portal.dbmi.hms.harvard.edu/projects/n2c2-nlp/). We used REDCap 12.4.31 to collect physician responses.
Data analysis	This work uses several open-source libraries including HuggingFace Transformers 4.19.2, Datasets, 2.2.2, Evaluate 0.1.1, wandb 0.12.17, matplotlib 3.5.2, seaborn 0.12.2, pandas 1.4.2, ray 2.0.0, sklearn 1.1.1, deepspeed 0.8.0+384f17b, NVidia Apex, XGBoost 1.6.1 and spaCy 3.5.0. Our experimental framework involves the utilization of these libraries and in some cases modification of them. We will release code to replicate the pretraining, finetuning and testing of the models described in this paper at the time of publication. We included detailed methods and implementation steps in the Methods and Supplementary Information to allow for independent replication.<br><br>Code for experiment: https://github.com/nyuolab/NYUTron<br>Code for preprocessing i2b2-2012: https://github.com/nyuolab/i2b2_2012_preprocessing

For manuscripts utilizing custom algorithms or software that are central to the research but not yet described in published literature, software must be made available to editors and reviewers. We strongly encourage code deposition in a community repository (e.g. GitHub). See the Nature Portfolio guidelines for submitting code & software for further information.

Data

Policy information about availability of data

All manuscripts must include a data availability statement. This statement should provide the following information, where applicable:

- Accession codes, unique identifiers, or web links for publicly available datasets
- A description of any restrictions on data availability
- For clinical datasets or third party data, please ensure that the statement adheres to our policy

We provide links for the following public data:
MIMIC-III dataset (https://physionet.org/content/mimiciii/1.4/).
i2b2-2012 NER dataset (https://portal.dbmi.hms.harvard.edu/projects/n2c2-nlp/).

The clinical data used for the pretraining, finetuning, validation, and test sets were collected from the NYU Langone Health System EHR maintained by the NYULH Datacore team. Text data was stripped of rich text features and directly included in the dataset "as-is", and was augmented with structured features where noted. It consists of the production medical records of NYU Langone and cannot be made publicly available.

Human research participants

Policy information about studies involving human research participants and Sex and Gender in Research.

Reporting on sex and gender	We did not perform sex and gender analysis
Population characteristics	We performed analysis of model performance on test sets that are stratified by race and age. Our pretraining population has a mean age of 50.66 with standard deviation of 28.52. Our pretraining population has a self-reporting male-female ratio of 3:4. Our finetuning population has a mean age of 49.17 and a standard deviation of 28.76. Our finetuning population has a self-reporting male-female ratio of 3:4.
Recruitment	We recruit anyone who is admitted to the NYU Langone Health Hospital from 2011 to 2022 April.
Ethics oversight	NYU Langone Health Institutional Review Board

Note that full information on the approval of the study protocol must also be provided in the manuscript.

Field-specific reporting

Please select the one below that is the best fit for your research. If you are not sure, read the appropriate sections before making your selection.

☒ Life sciences ☐ Behavioural & social sciences ☐ Ecological, evolutionary & environmental sciences

For a reference copy of the document with all sections, see nature.com/documents/nr-reporting-summary-flat.pdf

Life sciences study design

All studies must disclose on these points even when the disclosure is negative.

Sample size	For pretraining, we used 7,247,694 notes with 387,144 patients. The notes were from 2011 to 2020.<br>For finetuning, we used 506,740 notes with 413,845 patients. The notes were from 2011 to 2021.<br>Each clinical note is considered a note sample and each patient id is considered a patient sample. The sample size is determined by counting the number of notes or patients in our dataset. Our goal was to build a dataset at the scale of our health system, and the sample sizes were determined by the size of the NYU Langone EHR. We performed additional scaling experiments in the manuscript to investigate effects of smaller datasets.
Data exclusions	For both pretraining and finetuning, we excluded notes that are not signed by medical professionals (physicians, residents, physician assistants, nurse practitioners, fellows). This is because other types of notes (e.g., from pastor and social workers) do not record the clinical decision making process. For finetuning, we excluded discharge notes from the rehabilitation, dialysis, and palliative care departments because these are not acute care admissions.
Replication	For retrospective study, we ran experiments using 5 different random seeds and achieve similar results.<br>The prospective study was run in a live clinical environment, inference results were run once and then served to clinicians via e-mail.
Randomization	We split the pretraining data in 3 splits with ratio 8:1:1. We split the finetuning data into 4 sets: training, validation, test, and temporal test set. The first 3 sets are notes from January 2011 to May 2021, with a ratio of 8:1:1. The temporal test set are notes from June to December of 2021.
Blinding	For all experiments, at test time, investigators were blinded to group allocation at time of analysis.

Reporting for specific materials, systems and methods

We require information from authors about some types of materials, experimental systems and methods used in many studies. Here, indicate whether each material, system or method listed is relevant to your study. If you are not sure if a list item applies to your research, read the appropriate section before selecting a response.

Materials & experimental systems

n/a	Involved in the study
☒	☐ Antibodies
☒	☐ Eukaryotic cell lines
☒	☐ Palaeontology and archaeology
☒	☐ Animals and other organisms
☐	☒ Clinical data
☒	☐ Dual use research of concern

Methods

n/a	Involved in the study
☒	☐ ChIP-seq
☒	☐ Flow cytometry
☒	☐ MRI-based neuroimaging

Clinical data

Policy information about clinical studies

All manuscripts should comply with the ICMJE guidelines for publication of clinical research and a completed CONSORT checklist must be included with all submissions.

Clinical trial registration	Did not register prospectively.
Study protocol	NYU Langone Health Medical Center Information Technology (https://nyulangone.org)
Data collection	Data was collected from NYU Langone Health System prospectively for all patients encountered in the health system for the duration of the trial.
Outcomes	Our primary outcome was successful identification of all-cause 30-day readmissions at discharge during the index admission.

March 2021
